# Identification of integration site library for the development of plasmid-free microbial cell factory in *Bacillus subtilis*

**DOI:** 10.1016/j.synbio.2026.04.042

**Published:** 2026-06-29

**Authors:** Zhen Zong, Yongting Luo, Wan-Qing Yuan, Feng-Ping Xu, Jia-Jun Zhu, Wei Chen, Wen-Wen Zhou

**Affiliations:** a Institute of Food Bioscience and Technology, College of Biosystems Engineering and Food Science, Zhejiang University, Hangzhou, Zhejiang, 310058, China; b Department of Nutrition and Health, China Agricultural University, Beijing, 100193, China

**Keywords:** *Bacillus subtilis*, Integration sites, Metabolic engineering, Secretory protein, Shuttle vector

## Abstract

*Bacillus subtilis* was widely used for enzyme production and was gradually engineered to biosynthesize value-added chemicals with the development of genetic parts for it. Compared to other genetic parts for expression, the identified integration sites were fewer, which limits the development of *B. subtilis*. Here, a library of integration sites was developed for *B. subtilis*. All candidate sites were selected at the 3′-untranslated region of two opposite nonessential genes and separated by essential genes among genome to avoid the destruction for coding sequence of genes and eliminate the homologously recombined strains with the loss of essential genes. The expression of GFP and cell growth were detected for candidate sites to evaluate the gene expression strength and the influence on cell growth. As a result, 12 loci revealed higher gene expression level and cell growth compared with control site *amyE*, the highest expression site *spxA* was 1.89 times as high as *amyE*. Using the developed integration sites library, threefold gene expression range could be achieved without the replacement of promoter and RBS. When the integration site library was used to construct cell factories, the production of lacto-*N*-triose II and lycopene was increased by 95% and 83%, respectively. In addition, integration site library was also successfully used to increase the enzymatic activity of secretory β-galactosidase by 101% when the strain using *spxA* locus compared with that using *amyE.* The developed integration site library could accelerate the construction of stable and plasmid-free cell factories for *B. subtilis* in the future.

## Graphical abstract

**Figure:**
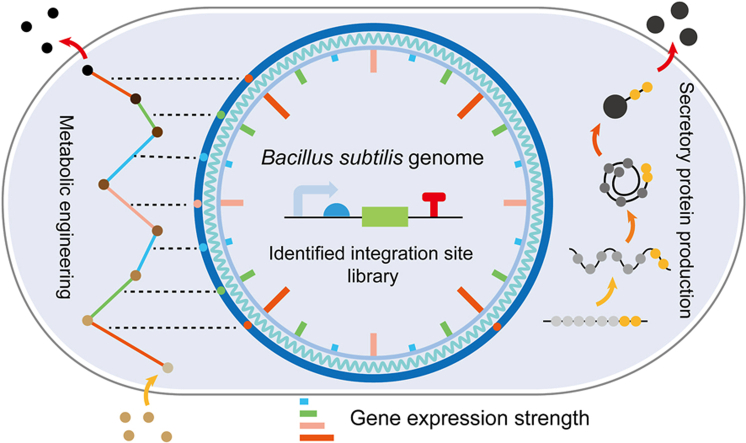

## 1 Introduction

*Bacillus subtilis* (*B. subtilis*), a Gram-positive bacterium, has been found for almost 200 years. As one of the earliest genome-sequenced bacteria [1], its genetic background and physiological properties were extensively investigated and it has been granted “Generally Recognized as Safe” (GRAS) status by the United States Food and Drug Administration (FDA) [2]. The most important application field of *B. subtilis* is the production of enzymes such as amylases, proteases, and lipases, owing to its superior ability for protein secretion [3]. In addition, *B. subtilis* is also known to be a major host for the production of vitamin B2 in the fermentation industry [4], and is being developed as an efficient chassis to produce other value-added chemicals with the progress of synthetic biology [5,6].

As a model bacterium with many applications, *B. subtilis* has attracted much interest in synthetic biology. Genetic parts including promoters, RBS, terminators, and replicons are the basic building blocks of synthetic biology and have been characterized and designed for the species *B. subtilis* [[7], [8], [9], [10], [11]]. These genetic parts support the development and application of plasmid-based gene expression systems [12,13]. Using plasmid-based expression systems, CRISPR-Cas9/Cas9 nickase/Cpf1 gene editing methods were gradually developed for *B. subtilis* to speed up the development of engineered strains and regulate gene expression [[14], [15], [16]]. The expression of target gene in plasmid, however, not only required additional antibiotics to sustain the replication but also increased the genetic instability of engineered strains during fermentation. An alternative method for gene expression is to integrate expression cassette of target gene into genome, which is a suitable approach for creating stable strains and widely used in *Saccharomyces cerevisiae* (*S. cerevisiae*) and *Escherichia coli* (*E. coli*) [[17], [18], [19], [20]]. Many integration sites have been developed for model microorganisms like *S. cerevisiae* and *E. coli* to accommodate the integration of target genes [17,[21], [22], [23]]. However, the integration sites identified for *B. subtilis* were fewer than those for other model microorganisms and the related research was also delayed compared with that for other genetic parts.

To date, most of the identified integration sites for *B. subtilis* were designed by destroying the regions of coding sequence (CDS) of nonessential genes such as *amyE*, *lacA*, *sacA*, and *thrC* [24], which may restrict the engineered strain for the utilization of carbon source or the biosynthesis of amino acid and further impairing the robustness of the engineered strain. Therefore, the development of additional integration sites without destroying the CDS is necessary to build *B. subtilis* microbial cell factories with greater resilience and maintains its capability for utilizing natural resources.

In this study, a stable integration site library was developed for *B. subtilis*, the integration sites were selected at the 3′-untranslated region (3′-UTR) of two apposite endogenous genes and separated by essential genes to avoid the destruction for gene CDS and maintain the stability of engineered strains. As a result, a number of integration sites revealed advantages over reported sites in terms of gene expression and cell growth. These identified integration sites could be used to develop stable and efficient *B. subtilis* cell factories to product value-added chemicals and secretory proteins.

## 2 Materials and methods

### 2.1 Strains, medium and reagents

All strains used in this study were listed in Table 1. *E. coli* DH5α or BL21(DE3) were used for plasmid construction. *B. subtilis* 168 was employed for the expression of GFP and the production of LNTII, lycopene, and β-galactosidase. Both of *E. coli* and *B. subtilis* 168 were cultured in Luria Bertani (LB) medium (10 g/L NaCl, 10 g/L peptone, and 5 g/L yeast extract). The fermentation medium using for the production of lycopene contained: 6 g/L peptone, 12 g/L yeast extract, 60 g/L glucose, 6 g/L (NH4)_2_SO_4_, 12 g/L K_2_HPO_4_·3H_2_O, 2.5 g/L KH_2_PO_4_, 3 g/L MgSO_4_·7H_2_O, 1.5 g/L Urea. For the production of LNTII, 20 g/L lactose was added to the fermentation medium. LB medium was used for the production of β-galactosidase. If necessary, spectinomycin (50 μg/mL) was added for plasmids construction. Chloramphenicol (5 μg/mL) or spectinomycin (100 μg/mL) was added for the screening of *B. subtilis* transformants. Takara PrimeSTAR® Max DNA Polymerase was used for PCR. Plasmid and gel extraction kits were purchased from Yeasen (Yeasen, Shanghai, China), Smart Assembly Cloning Kit (Smart-Lifescience, Changzhou, China) was used for the construction of plasmids. LNTII and lycopene standards were purchased from Yuanye (Yuanye, Shanghai, China) and Solarbio (Solarbio, Beijing, China), respectively. β-galactosidase Assay Kit (Beyotime Biotechnology, Shanghai, China) and *o*-nitrophenol (Aladdin, Shanghai, China) were used to determine the enzymatic activity of secretory β-galactosidase.

**Table 1:** Strains used in this study.

Strains	Description	References
DH5α	F^−^ φ80lacZΔM15Δ(lacZYA-argF) U169, recA1 endA1 hsdR17 (r_k_^−^ m_k_^+^) phoA supE44 thi-1 gyrA96 relA1	Lab stock
BL21(*DE3*)	F− ompT hsdSB (rB− mB−) gal dcm rne131 λ (DE3)	Lab stock
*B. subtilis* 168	trpC2	Lab stock
BS-*amyE*-GFP	*B. subtilis* 168, Δ*amyE*::Pveg-GFP-rrnBT1T2	This study
BS-*spxA*-GFP	*B. subtilis* 168, Pveg-GFP-rrnBT1T2 was integrated in the downstream region of *spxA* CDS	This study
BS-*tatCY*-GFP	*B. subtilis* 168, Pveg-GFP-rrnBT1T2 was integrated in the downstream region of *tatCY* CDS	This study
BS-*rlmCD*-GFP	*B. subtilis* 168, Pveg-GFP-rrnBT1T2 was integrated in the downstream region of *rlmCD* CDS	This study
BS-*abrB*-GFP	*B. subtilis* 168, Pveg-GFP-rrnBT1T2 was integrated in the downstream region of *abrB* CDS	This study
BS-*pchA*-GFP	*B. subtilis* 168, Pveg-GFP-rrnBT1T2 was integrated in the downstream region of *pchA* CDS	This study
BS-*yybC*-GFP	*B. subtilis* 168, Pveg-GFP-rrnBT1T2 was integrated in the downstream region of *yybC* CDS	This study
BS-*yneQ*-GFP	*B. subtilis* 168, Pveg-GFP-rrnBT1T2 was integrated in the downstream region of *yneQ* CDS	This study
BS-*rsbRB*-GFP	*B. subtilis* 168, Pveg-GFP-rrnBT1T2 was integrated in the downstream region of *rsbRB* CDS	This study
BS-*yhdI*-GFP	*B. subtilis* 168, Pveg-GFP-rrnBT1T2 was integrated in the downstream region of *yhdI* CDS	This study
BS-*nupG*-GFP	*B. subtilis* 168, Pveg-GFP-rrnBT1T2 was integrated in the downstream region of *nupG* CDS	This study
BS-*yqgY*-GFP	*B. subtilis* 168, Pveg-GFP-rrnBT1T2 was integrated in the downstream region of *yqgY* CDS	This study
BS-*ydfD*-GFP	*B. subtilis* 168, Pveg-GFP-rrnBT1T2 was integrated in the downstream region of *ydfD* CDS	This study
BS-*opuD*-GFP	*B. subtilis* 168, Pveg-GFP-rrnBT1T2 was integrated in the downstream region of *opuD* CDS	This study
BS-*ykrA*-GFP	*B. subtilis* 168, Pveg-GFP-rrnBT1T2 was integrated in the downstream region of *ykrA* CDS	This study
BS-*psmB*-GFP	*B. subtilis* 168, Pveg-GFP-rrnBT1T2 was integrated in the downstream region of *psmB* CDS	This study
BS-*ydbO*-GFP	*B. subtilis* 168, Pveg-GFP-rrnBT1T2 was integrated in the downstream region of *ydbO* CDS	This study
BS-*aldX*-GFP	*B. subtilis* 168, Pveg-GFP-rrnBT1T2 was integrated in the downstream region of *aldX* CDS	This study
BS-*racA*-GFP	*B. subtilis* 168, Pveg-GFP-rrnBT1T2 was integrated in the downstream region of *racA* CDS	This study
BS-*ymaD*-GFP	*B. subtilis* 168, Pveg-GFP-rrnBT1T2 was integrated in the downstream region of *ymaD* CDS	This study
BS-*hlpB*-GFP	*B. subtilis* 168, Pveg-GFP-rrnBT1T2 was integrated in the downstream region of *hlpB* CDS	This study
BS-*gerBC*-GFP	*B. subtilis* 168, Pveg-GFP-rrnBT1T2 was integrated in the downstream region of *gerBC* CDS	This study
BS-*pdaC*-GFP	*B. subtilis* 168, Pveg-GFP-rrnBT1T2 was integrated in the downstream region of *pdaC* CDS	This study
BS-*mtnD*-GFP	*B. subtilis* 168, Pveg-GFP-rrnBT1T2 was integrated in the downstream region of *mtnD* CDS	This study
BS-*ilvE*-GFP	*B. subtilis* 168, Pveg-GFP-rrnBT1T2 was integrated in the downstream region of *ilvE* CDS	This study
BS-*spoVM*-GFP	*B. subtilis* 168, Pveg-GFP-rrnBT1T2 was integrated in the downstream region of *spoVM* CDS	This study
BS-*rlmI*-GFP	*B. subtilis* 168, Pveg-GFP-rrnBT1T2 was integrated in the downstream region of *rlmI* CDS	This study
BS-*yfkD*-GFP	*B. subtilis* 168, Pveg-GFP-rrnBT1T2 was integrated in the downstream region of *yfkD* CDS	This study
BS-*cotN*-GFP	*B. subtilis* 168, Pveg-GFP-rrnBT1T2 was integrated in the downstream region of *cotN* CDS	This study
BS-*yoaH*-GFP	*B. subtilis* 168, Pveg-GFP-rrnBT1T2 was integrated in the downstream region of *yoaH* CDS	This study
BS-*kduD*-GFP	*B. subtilis* 168, Pveg-GFP-rrnBT1T2 was integrated in the downstream region of *kduD* CDS	This study
BS-*fhuD*-GFP	*B. subtilis* 168, Pveg-GFP-rrnBT1T2 was integrated in the downstream region of *fhuD* CDS	This study
BS-*rsbRC*-GFP	*B. subtilis* 168, Pveg-GFP-rrnBT1T2 was integrated in the downstream region of *rsbRC* CDS	This study
BS-*guaC*-GFP	*B. subtilis* 168, Pveg-GFP-rrnBT1T2 was integrated in the downstream region of *guaC* CDS	This study
BS-*ytvI*-GFP	*B. subtilis* 168, Pveg-GFP-rrnBT1T2 was integrated in the downstream region of *ytvI* CDS	This study
BS-*ninI*-GFP	*B. subtilis* 168, Pveg-GFP-rrnBT1T2 was integrated in the downstream region of *ninI* CDS	This study
BS-*ansR*-GFP	*B. subtilis* 168, Pveg-GFP-rrnBT1T2 was integrated in the downstream region of *ansR* CDS	This study
BS-*lacA*-GFP	*B. subtilis* 168, Δ*lacA*::Pveg-GFP-rrnBT1T2	This study
BS-*thrC*-GFP	*B. subtilis* 168, Δ*thrC*::Pveg-GFP-rrnBT1T2	This study
BS-*sacA*-GFP	*B. subtilis* 168, Δ*sacA*::Pveg-GFP-rrnBT1T2	This study
BS-*gltA*-GFP	*B. subtilis* 168, Δ*gltA*::Pveg-GFP-rrnBT1T2	This study
BS-*pyrD*-GFP	*B. subtilis* 168, Δ*pyrD*::Pveg-GFP-rrnBT1T2	This study
BS-*ninI*-Pzwf-RBS0-GFP	*B. subtilis* 168, Pzwf-RBS0-GFP-rrnBT1T2 was integrated in the downstream region of *ninI* CDS	This study
BS-*ninI*-PlepA-RBS0-GFP	*B. subtilis* 168, PlepA-RBS0-GFP-rrnBT1T2 was integrated in the downstream region of *ninI* CDS	This study
BS-*ninI*-Pveg-RBS4-GFP	*B. subtilis* 168, Pveg-RBS4-GFP-rrnBT1T2 was integrated in the downstream region of *ninI* CDS	This study
BS-*ninI*-Pveg-RBS7-GFP	*B. subtilis* 168, Pveg-RBS7-GFP-rrnBT1T2 was integrated in the downstream region of *ninI* CDS	This study
BSLNTII-1	*B. subtilis* 168, Δ*amyE*::Pveg-*NmlgtA*-rrnBT1T2, Pveg-*EclacY*-rrnBT1T2 was integrated in the downstream region of *guaC* CDS	This study
BSLNTII-2	*B. subtilis* 168, Pveg-*NmlgtA*-rrnBT1T2 was integrated in the downstream region of *abrB* CDS, Pveg-*EclacY*-rrnBT1T2 was integrated in the downstream region of *guaC* CDS	This study
BSLNTII-3	*B. subtilis* 168, Δ*amyE*::Pveg-*NmlgtA*-rrnBT1T2, Pveg-*NmlgtA*-rrnBT1T2 was integrated in the downstream region of *abrB* CDS, Pveg-*EclacY*-rrnBT1T2 was integrated in the downstream region of *guaC* CDS	This study
BSLycopene-1	*B. subtilis* 168, Pveg-*PacrtE*-rrnBT1T2 was integrated in the downstream region of *ydfD* CDS, Pveg-*PagcrtB*-rrnBT1T2 was integrated in the downstream region of *psmB* CDS, Pveg-*BtcrtI*-rrnBT1T2 was integrated in the downstream region of *aldX* CDS	This study
BSLycopene-2	*B. subtilis* 168, Pveg-*PacrtE*-rrnBT1T2 was integrated in the downstream region of *spxA* CDS, Pveg-*PagcrtB*-rrnBT1T2 was integrated in the downstream region of *psmB* CDS, Pveg-*BtcrtI*-rrnBT1T2 was integrated in the downstream region of *aldX* CDS	This study
BSLycopene-3	*B. subtilis* 168, Pveg-*PacrtE*-rrnBT1T2 was integrated in the downstream region of *ydfD* CDS, Pveg-*PagcrtB*-rrnBT1T2 was integrated in the downstream region of *tatCY* CDS, Pveg-*BtcrtI*-rrnBT1T2 was integrated in the downstream region of *aldX* CDS	This study
BSLycopene-4	*B. subtilis* 168, Pveg-*PacrtE*-rrnBT1T2 was integrated in the downstream region of *ydfD* CDS, Pveg-*PagcrtB*-rrnBT1T2 was integrated in the downstream region of *psmB* CDS, Pveg-*BtcrtI*-rrnBT1T2 was integrated in the downstream region of *abrB* CDS	This study
BSLycopene-5	*B. subtilis* 168, Pveg-*PacrtE*-rrnBT1T2 was integrated in the downstream region of *spxA* CDS, Pveg-*PagcrtB*-rrnBT1T2 was integrated in the downstream region of *tatCY* CDS, Pveg-*BtcrtI*-rrnBT1T2 was integrated in the downstream region of *aldX* CDS	This study
BSLycopene-6	*B. subtilis* 168, Pveg-*PacrtE*-rrnBT1T2 was integrated in the downstream region of *ydfD* CDS, Pveg-*PagcrtB*-rrnBT1T2 was integrated in the downstream region of *tatCY* CDS, Pveg-*BtcrtI*-rrnBT1T2 was integrated in the downstream region of *abrB* CDS	This study
BSLycopene-7	*B. subtilis* 168, Pveg-*PacrtE*-rrnBT1T2 was integrated in the downstream region of *spxA* CDS, Pveg-*PagcrtB*-rrnBT1T2 was integrated in the downstream region of *psmB* CDS, Pveg-*BtcrtI*-rrnBT1T2 was integrated in the downstream region of *abrB* CDS	This study
BS-*EclacZ*-*amyE*	*B. subtilis* 168, Pveg-SPphoB-*EclacZ*-rrnBT1T2 was integrated in the downstream region of *amyE* CDS	This study
BS-*EclacZ*-*abrB*	*B. subtilis* 168, Pveg-SPphoB-*EclacZ*-rrnBT1T2 was integrated in the downstream region of *abrB* CDS	This study
BS-*EclacZ*-*tatCY*	*B. subtilis* 168, Pveg-SPphoB-*EclacZ*-rrnBT1T2 was integrated in the downstream region of *tatCY* CDS	This study
BS-*EclacZ*-*spxA*	*B. subtilis* 168, Pveg-SPphoB-*EclacZ*-rrnBT1T2 was integrated in the downstream region of *spxA* CDS	This study

### 2.2 Plasmids construction

All primers and plasmids used in this study were listed in Table S1 and Table S2, respectively. The DNA sequences of the genes (*NmlgtA*, GenBank: AAF42258.1; *PacrtE*, GenBank: BAA14124.1; *PagcrtB*, GenBank: AAA64982.1; *BtcrtI*, GenBank: AAX20903.1) were codon-optimized and synthesized by Sangon Biotech (Shanghai, China). *EclacY* (GenBank: NP_414877.1) and *EclacZ* (GenBank: AAA24053.1) were amplified from *E. coli* genome. The secretion signal peptide SPphoB was amplified from *B. subtilis* genome. All plasmids were constructed using Gibson assembly method. To construct site integration plasmids for GFP, about 700 bp homologous arms for candidate integration sites were amplified from *B. subtilis* genome (the upstream homologous arms of *tatCY* and *abrB* sites were 400 bp and 300 bp respectively) and recombined with GFP expression cassette, chloramphenicol resistance and shuttle plasmid backbone which were amplified from the plasmid pDG1662-loxp-Pveg-GFP-rrnBT1T2 (Supplementary Table S2). The homologous arms amplified for testing *lacA*, *thrC*, *sacA*, *gltA*, and *pyrD* referred the reports [24,25]. The promoters Pzwf and PlepA were amplified from *B. subtilis* genome. To construct sites integration plasmids for *NmlgtA*, *EclacY*, *PacrtE*, *PagcrtB*, *BtcrtI*, and *EclacZ*, the empty plasmids for related integration sites were constructed firstly which replaced GFP CDS with *Bsa*I restriction site, and then these empty plasmids were linearized by *Bsa*I and recombined with the target genes. To construct the optimized plasmids for secretory proteins, pSC101 replicon was amplified from pKD46 and recombined with the backbone of empty plasmids without spectinomycin resistance gene for *B. subtilis*.

### 2.3 Strains construction

*B. subtilis* competent cell was prepared according to the report [7]. For the integration of target genes into *B. subtilis* chromosome, 500 ng integration plasmid were added in competent cell and incubated at 37 °C for 1.5 h, then spread in LB plates contains 5 mg/L chloramphenicol. Clone PCR was used to verify integration. The helper plasmid pDR244Cre was transformed to integrated strain and the strain was cultured at 30 °C for 36 h to sufficiently express Cre recombinase and remove chloramphenicol selection marker. Then the PCR verified strain was cultured in antibiotic-free LB medium at 37 °C for 48 h to eliminate helper plasmid.

### 2.4 Fluorescence and biomass determination

The constructed strains used for the evaluation of candidate integration sites were cultured in 2 mL LB overnight at 37 °C, then seed culture was transferred 200 μL to 100 mL shake flask with 20 mL LB and cultured for 24 h at 37 °C. For the determination of fluorescence intensity of GFP, 100 μL cell culture was injected in 96-well black plate and measured by microplate reader (Agilent BioTek Synergy H1). Fluorescence intensity of GFP was measured at an excitation wavelength of 490 nm and an emission wavelength of 530 nm with a gain value 50. The optical density of cells at 600 nm was measured by UV-Vis spectrophotometer to indicate the biomass. Relative fluorescent units (RFU) were defined as the fluorescence intensity of GFP for per OD_600_ (Fluorescence intensity _GFP_/OD_600_).

### 2.5 Shake flask fermentation

For the production of LNTII and lycopene, the engineered strains were cultured overnight in 2 mL LB at 37 °C, and then seed culture was transferred to 100 mL shake flask with 20 mL fermentation medium with initial OD_600_ of 0.1 and fermented for 48 h for LNTII and 72 h for lycopene at 37 °C. To produce β-galactosidase, the engineered strains were cultured overnight in 2 mL LB at 37 °C, and then 1% (v/v) seed culture was transferred to 100 mL shake flask with 20 mL LB medium and fermented for 48 h. All fermentation experiments were performed in triplicate.

### 2.6 Sample preparation and analysis

The procedure of sample preparation for LNTII referred the previous reported method [26]. High performance liquid chromatography (HPLC, Waters 2695) equipped with an evaporative light scattering detector (ELSD, Waters 2424) and an amide column (250 mm × 4.6 mm, 0.50 μm) was used for the qualitative and qualitative analysis of LNTII, and detail parameter of HPLC were set same to the detection for glucose [27].

500 μl lycopene-fermented culture was centrifugated at 12000 rpm for 5 min to harvest cell precipitate, and then equal volume acetone with 1% butylated hydroxytoluene was mixed with cell precipitate to extract intracellular lycopene as described previously [28]. The qualitative and quantitative analysis of lycopene was performed using ultrahigh-performance liquid chromatography (UHPLC, Thermo Scientific Dionex Ultimate 3000) equipped with a C18 column (250 mm × 4.6 mm, 0.50 μm). Detail parameters setting for HPLC referred the previous method [29].

### 2.7 β-galactosidase activity analysis

To determine the enzymatic activity of secretory β-galactosidase, 500 μL β-galactosidase-fermented culture was centrifugated at 12000 rpm for 5 min and 10 μL supernatant was mixed with 40 μL ddH_2_O and 50 μL reaction reagent and incubated at 37 °C for 30 min. After the incubation, 150 μL termination reagent was added in samples to determine the optical density of the reaction product *o*-nitrophenol at 420 nm and quantify the content according to the *o*-nitrophenol standard curve. The enzymatic activity of secretory β-galactosidase (U) was defined as the content of *o*-nitrophenol produced by 1 mL supernatant at 1 min (1U = 1 nmol *o*-nitrophenol/min/mL).

## 3 Results and discussion

### 3.1 Construction of integration site library for *Bacillus subtilis*

**Fig. 1:**
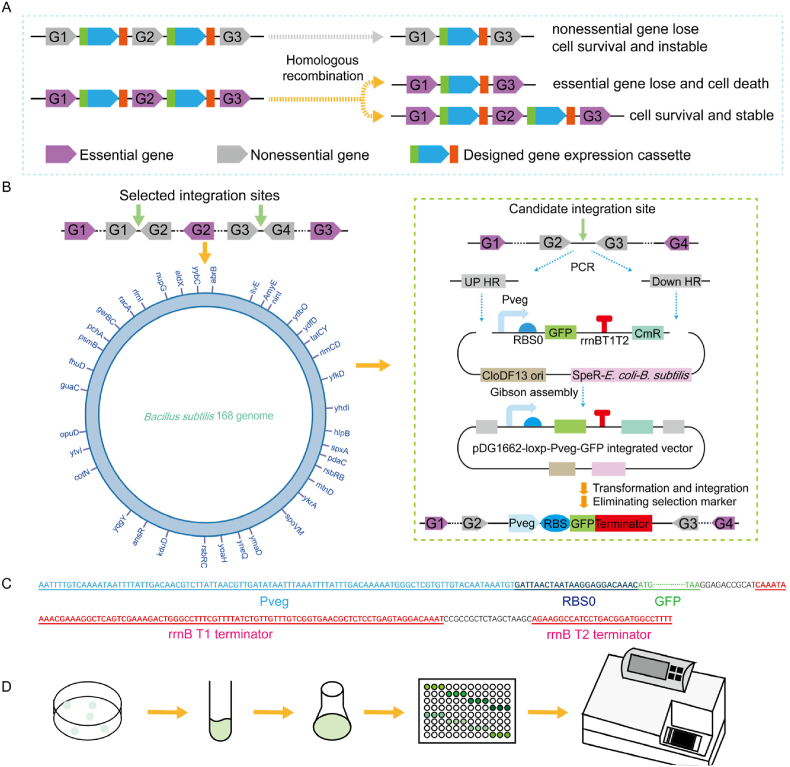
The schematic of selection principle and testing workflow for candidate integration sites. (A) Different results caused by homologous recombination between two integration sites inserting repeat sequences. (B) Distribution of candidate integration sites in genome and the construction process of testing strains for candidate integration sites. (C) The composition of GFP report system. (D) Testing process of constructed strain for selected integration sites.

The selection of suitable candidate integration sites should take into account the stability of the integrated genes and the effect on endogenous genes. When repeat promoter, CDS or terminator were used to express target genes at two or more integration loci, the homologous recombination-mediated allele replacement may occur at these loci, increasing the instability of engineered strains. To ensure the stable utilization of multiple integration loci, the candidate integration loci were selected among essential genes which could auto-eliminate the unstable strain due to the loss of essential gene (Fig. 1A) [30]. Furthermore, to avoid the destruction of integrated genes for endogenous genes and minimize the impact on the expression of essential genes, the candidate integration loci were further selected at the 3′-UTR regions of two opposite nonessential genes (Fig. 1B). Therefore, an integration site library was constructed according to the above features which includes 36 candidates across the genome. To assess the performance of the integration site library, green fluorescent protein (GFP) was integrated in candidate loci and expressed under the control of the strong constitutive promoter Pveg (Fig. 1C) [7]. For the analysis of gene expression levels and cell growth, relative fluorescence intensities (RFU), and OD_600_ were determined and the common integration site *amyE* was used as control (Fig. 1D).

**Fig. 2:**
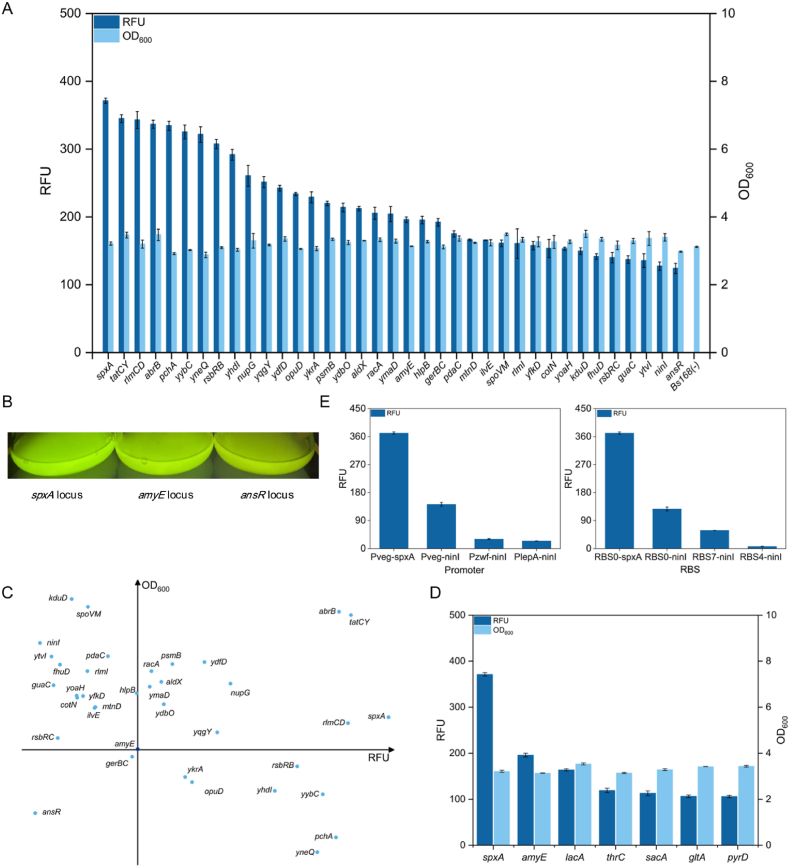
Comprehensive assessment for integration sites library. (A) The relative fluorescence intensity of GFP and cell growth condition of constructed strains for candidate integration sites. The common integration site *amyE* was used as the positive control and *B. subtilis* 168 without integrating GFP was used as the blank control. (B) Fluorescence performance of GFP-expressed strains using *spxA*, *amyE*, and *ansR* loci respectively. (C) Two-dimensional coordinate system constructed for the display of performance of candidate integration sites. The common integration site *amyE* was set as the coordinate origin, and integration sites were divided into four categories according to RFU and OD_600_. (D) The performance comparison between *spxA* and the other reported integration sites. (E) Synergistic results of the integration site incorporated with promoter/RBS design for gene expression. The tested promoters (Pzwf and PlepA) and RBSs (RBS4 and RBS7) were weaker than Pveg and RBS0, respectively. All synergistic outcomes tested in the identified integration site *ninI*.

As shown in Fig. 2A, all candidate loci could be used to integrate and express GFP, and the relative fluorescence intensity and OD_600_ revealed significant differences among the candidate loci. Nineteen candidate loci revealed higher RFU compared with *amyE* locus, the highest RFU occurred in *spxA* locus which was 1.89 times as high as *amyE* locus, and the growth of the related strain wasn't affected by the higher GFP expression compared to the strain expressing GFP in *amyE* locus. Seventeen candidate loci revealed lower RFU compared with *amyE* locus, and *ansR* locus shown the lowest RFU which was only 63.3% of that expressed in *amyE* locus (Fig. 2B). Both the level of gene expression and growth statue of strains were important evaluation parameters for the integration sites. Therefore, a two-dimensional coordinate system was constructed using RFU and OD_600_ as X and Y axes to visually compare the performance of the candidate integration sites with that of the *amyE* locus. As shown in Fig. 2C, among the selected loci, 12 loci (*spxA*, *tatCY*, *rlmCD*, *abrB*, *nupG*, *yqgY*, *ydfD*, *psmB*, *ydbO*, *aldX*, *racA*, and *ymaD*) not only revealed higher RFU but achieved higher cell density compared to *amyE* locus, and these loci were appropriate for the integration of genes which require the relatively high expression. Compared to the *amyE* site, the integrated site *hlpB* had similar RFU but revealed a higher cell density and could be used as an alternative to the *amyE* site for gene integration and expression. Besides, 14 loci (*pdaC*, *mtnD*, *ilvE*, *spoVM*, *rlmI*, *yfkD*, *cotN*, *yoaH*, *kduD*, *fhuD*, *rsbRC*, *guaC*, *ninI*, and *ytvI*) revealed lower RFU but achieved higher cell density compared with *amyE* locus, and these loci were suitable for gene integration when relatively low expression is needed. The rest nine loci revealed lower cell density, which means the cell growth would be inhibited when target gene was integrated in these sites.

To further evaluate the expression level of the identified locus *spxA* among reported integration sites for *B. subtilis*, five additional reported integration sites *lacA*, *thrC*, *sacA*, *gltA*, and *pyrD* [24,25] were selected for comparison. The result shown that the gene expression level of the identified locus *spxA* was higher than all tested control sites, and this identified site had the potential for gene high expression in genome (Fig. 2D).

Using the identified integration site library, a 3-fold gene expression range could be achieved without changing the promoter and RBS, and the gene expression range could be further extended to 15.3-fold or 54.6-fold by incorporating the identified integration site with another promoter (PlepA) or RBS (RBS4) [7] respectively (Fig. 2E). The identified integration site library could be used not only for the production of secretory proteins by balancing the production of target proteins and cell growth but for metabolic engineering to improve the production of valuable compounds by modulating metabolic network and flux.

### 3.2 Construction of *B. subtilis* cell factories for the production of valuable chemicals using identified integration sites

*B. subtilis*, one of the most common microbial chassis, has been developed as cell factories to produce valuable chemicals. Both extracellular and intracellular molecules were selected for a comprehensive assessment of the availability of identified integration sites in the development of *B. subtilis* cell factories.

**Fig. 3:**
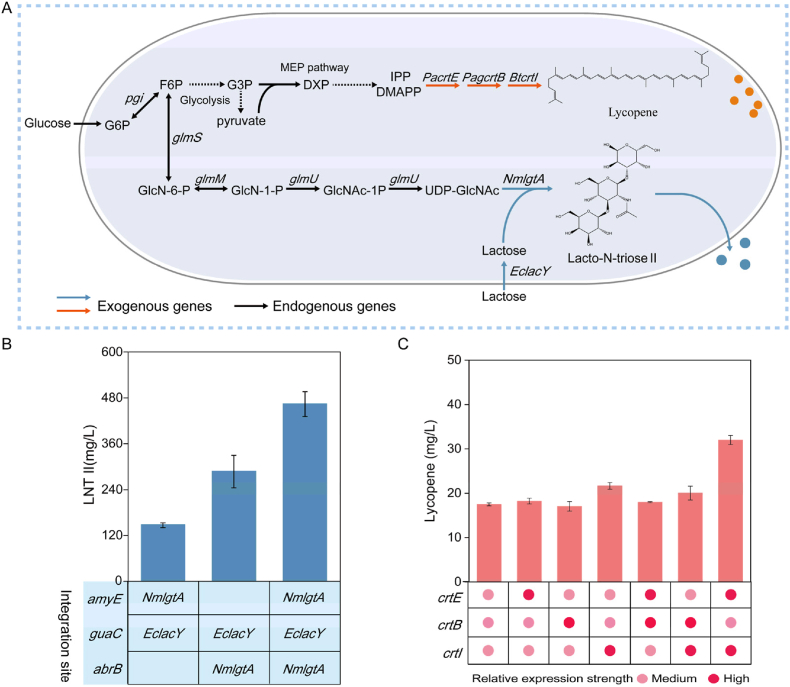
Construction of *B. subtilis* cell factories for the production of lacto-*N*-triose II and lycopene using identified integration sites. (A) The constructed biosynthetic pathway of lacto-*N*-triose II and lycopene in *B. subtilis*. *pgi*, glucose-6-phosphate isomerase; *glmS*, glucosamine-6-phosphate synthase; *glmM*, phosphoglucosamine mutase; *glmU*, N-acetylglucosamine-1-phosphate uridyltransferase/glucosamine-1-phosphate acetyltransferase; *NmlgtA*, β-1,3-*N*-acetylglucosaminyltransferase from *Neisseria meningitidis*; *EclacY*, lactose permease from *E. coli*; *PacrtE*, geranylgeranyl pyrophosphate synthase from *Pantoea ananatis*; *PagcrtB*, phytoene synthase from *Pantoea agglomerans*; *BtcrtI*, phytoene desaturase from *Blakeslea trispora*. (B) Overexpression of rate-limiting enzyme for the improved production of lacto-*N*-triose II using identified integration sites. (C) Modulation of biosynthetic pathway to increase the production of lycopene using identified integration sites.

Human milk oligosaccharides (HMOs), a complex class of oligosaccharides, have beneficial effects on the growth of infants [31]. Lacto-*N*-triose II (LNTII), the key precursor of lacto-*N*-tetraose and lacto-N-neotetraose (LNnT) [32], was selected as the target extracellular molecule to validate the feasibility of using identified integration sites to construct *B. subtilis* cell factories. For the production of LNTII, β-1,3-N-acetylglucosaminyltransferase (*lgtA*) and lactose permease (*lacY*) should be introduced in *B. subtilis* to transport lactose into cell and further condense it with UDP-*N*-acetylglucosamine to produce LNTII (Fig. 3A). Therefore, *lacY* from *E. coli* (*EclacY*) and *lgtA* from *Neisseria meningitidis* (*NmlgtA*) were integrated in *guaC* and *amyE* locus respectively, and expressed under the control of Pveg promoter. The resulting stain BSLNTII-1 produced 146.8 mg/L LNTII, indicating that the identified integration site *guaC* can be used in parallel with the common integration site *amyE*. *LgtA* was reported as a rate-limiting enzyme in LNTII biosynthesis and the overproduction of *LgtA* would significantly increase the production of LNnT [26]. To increase LNTII production, *NmlgtA* was shifted to *abrB* locus, a high expression integration site identified in our study, and the resulting strain BSLNTII-2 produced 287.1 mg/L LNTII, which was 1.95 times the level of BSLNTII-1. This result confirmed that the *abrB* locus can achieve higher expression of the integrated gene relative to the common integration site *amyE*. Multicopy integration of the key gene was a common strategy to overcome rate-limiting step of metabolic engineering. Therefore, another copy *NmlgtA* was integrated in the *amyE* locus of BSLNTII-2 to construct BSLNTII-3. The engineered strain BSLNTII-3 finally produced 463.6 mg/L LNTII, which was 2.1 times higher than that produced by BSLNTII-1. These engineered strains developed for LNTII indicated that the identified integration sites could be used to construct *B. subtilis* cell factories for the biosynthesis of extracellular products.

*B. subtilis* has an advantage over *E. coli* in the biosynthesis of terpenoids due to the existence of a strong precursor biosynthesis capability [33]. Lycopene, an intracellular metabolite, is a key precursor of carotenoids and has many benefits for human health [34]. Therefore, we engineered *B. subtilis* to produce lycopene using identified integration sites to evaluate the capability for the production of intracellular chemicals. For the biosynthesis of lycopene, three heterologous genes, geranylgeranyl pyrophosphate synthase (*crtE*), phytoene synthase (*crtB*), and phytoene desaturase (*crtI*) need to be introduced in *B. subtilis* to transform isopentenyl pyrophosphate (IPP) and dimethylallyl diphosphate (DMAPP) to lycopene. Therefore, *crtE* from *Pantoea ananatis* (*PacrtE*), *crtB* from *Pantoea agglomerans* (*PagcrtB*), and *crtI* from *Blakeslea trispora* (*BtcrtI*) were integrated in identified sites *ydfD*, *psmB*, and *aldX* respectively and expressed under the control of Pveg promoter to construct BSLycopene-1. The engineered strain BSLycopene-1 successfully synthesized lycopene with a titer of 17.4 mg/L.

In order to increase the productivity of the microbial cell factories designed to produce the target products, regulation of pathway gene expression is necessary to balance metabolic flux, especially in the multistep biosynthetic pathway. The identified integration site library can not only be used to integrate target genes but also to achieve differential expression of pathway genes to increase the production of target chemicals. To regulate the biosynthesis pathway of lycopene and the characteristic rate-limiting enzymes, three integration sites using in BSLycopene-1 were defined as medium expression strength sites (1.1∼1.2 fold of *amyE*) and the other three integration sites (*spxA*, *tatCY* and *abrB*) were selected and defined as high expression strength sites (1.7∼1.9 fold of *amyE*) to overexpress *PacrtE*, *PagcrtB*, and *BtcrtI* respectively. The genes involved in the biosynthesis of lycopene were overexpressed individually, resulting in strains BSLycopene-2, BSLycopene-3, and BSLycopene-4. As shown in Fig. 3C, lycopene production was improved to 21.6 mg/L when *BtcrtI* was integrated in high expression site *abrB*, and a slight improvement was also observed in the *PacrtE*-overexpressed strain. The overexpression of *PagcrtB* slightly reduced the yield of lycopene. Furthermore, three combinatorially overexpressed strains (*PacrtE* × *PagcrtB*, *PagcrtB* × *BtcrtI, PacrtE* × *BtcrtI*) were constructed. A synergistic effect was observed in the strain which co-overexpressed *PacrtE* and *BtcrtI*, with lycopene titer increasing further to 32 mg/L. The lycopene production in other two *PagcrtB*-overexpressed strains wasn't further improved. Consistent with the report [35], *crtE* and *crtI* were the rate-limiting enzymes and needed to be overexpressed to achieve the high yield of lycopene.

These engineered strains revealed the application potential of identified integration sites for the development of *B. subtilis* cell factories. The identified integration sites could serve as a complement to promoter, RBS, and other regulatory elements to modulate metabolic pathway, and be used in the metabolic engineering of *B. subtilis*.

### 3.3 Construction of *B. subtilis* cell factories for the production of secretory protein using identified integration sites

**Fig. 4:**
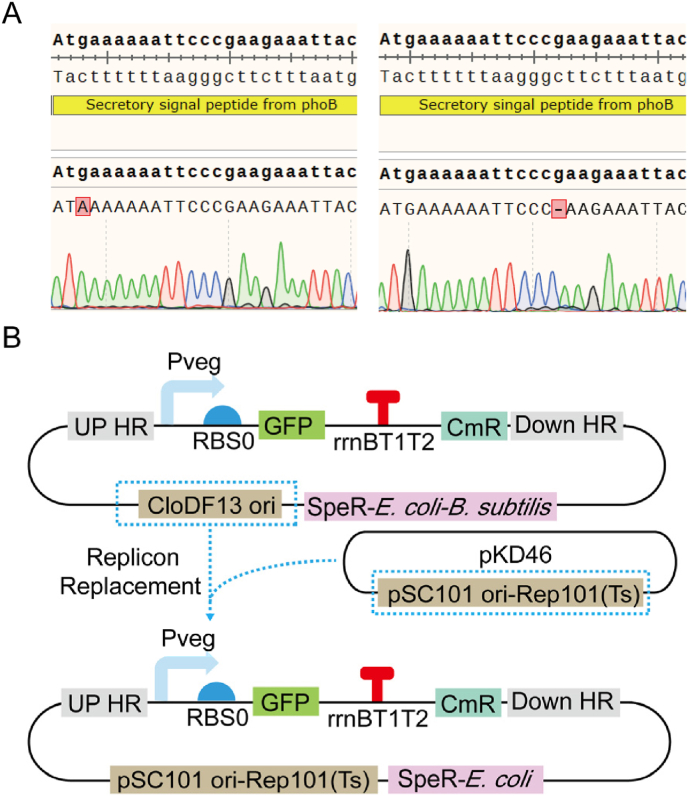
Construction of new shuttle vector with strong constitutive promoter for the production of secretory proteins in *B. subtilis*. (A) The mutation of secretory signal peptide in integrated plasmid for the production of secretory β-galactosidase. The strong constitute promoter Pveg with high transcription activity in *E. coli* was used to express secretory proteins in *B. subtilis*. (B) Schematic flow chart for the construction of new shuttle vector. The original replicon CloDF13 was replaced by low-copy-number replicon pSC101 and the spectinomycin resistance gene for *B. subtilis* was removed.

*B. subtilis* is well known for its protein secretory capacity and is widely used in the manufacture of industrial enzymes. In order to assess the compatibility of the identified integration sites for the biosynthesis of secretory proteins, *E. coli* β-galactosidase (*EclacZ*) was used as a reporter and enzymatic activity was determined. However, during the process of constructing an integration plasmid to express *EclacZ* with *PhoB* secretory signal peptide (SPphoB) using Pveg promoter, substitution or deletion mutations were occurred in the SPphoB that caused the termination of protein translation (Fig. 4A) and couldn't obtain the right plasmid. This result was similar to the report [36] that the construction of plasmid using strong constitutive promoter Pveg for secretory protein expression was mutated. This may be due to the strong transcriptional activity of Pveg in *E. coli*, leading to a translated peptide that competed with endogenous secretion proteins and ultimately caused cell death [37]. In our study, the integration vector contained a CloDF13 replicon with 20 copies that caused excessive production of secretory protein and led to cell death during the construction. To solve this problem, we replaced CloD13 with the pSC101 replicon (pSC101 ori with RepA101-ts, 5 copies) and removed the spectinomycin resistance gene for *B. subtilis*, thereby reducing growth burden for *E. coli* and the size of plasmid (Fig. 4B). When the reconstructed integration plasmid was used to express *EclacZ* with SPphoB using promoter Pveg, the required growth time for the transformants was extended to 24–36 h, but the sequencing validation of the recombinant plasmids was correct (Supplementary Fig. 1). This solution could be used for the construction of other *E. coli*-*B. subtilis* shuttle vectors with strong constitutive promoter recognized by *E. coli* and *B. subtilis*.

**Fig. 5:**
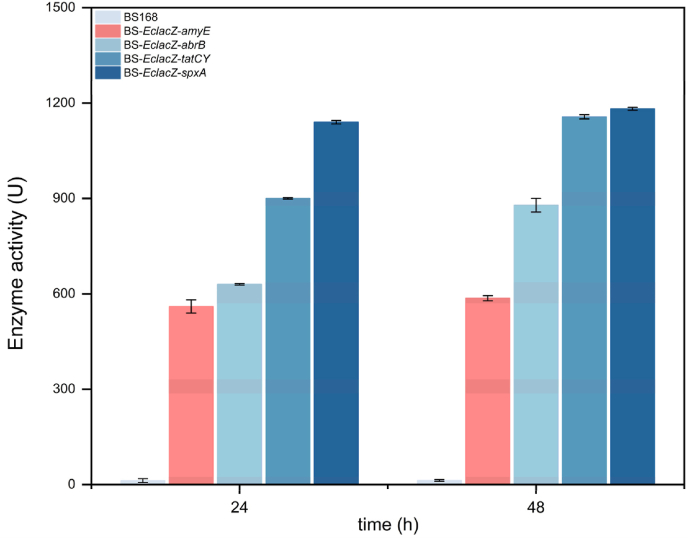
Construction of *B. subtilis* cell factories for the production of secretory β-galactosidase using identified integration sites. The β-galactosidase from *E. coli* with secretory signal peptide *PhoB* was expressed by Pveg and integrated in *amyE*, *abrB*, *tatCY*, and *spxA*, respectively. The constructed strains were cultured in LB medium for 48 h.

Three high expression integration sites (*spxA*, *tatCY* and *abrB*) were selected for the secretory production of β-galactosidase, combining the expression strength for inserted gene and the effects on cell growth. The enzymatic activity of supernatant was detected for secretory β-galactosidase after 48 h fermentation. As shown in Fig. 5, all three identified integration sites revealed higher enzymatic activity of β-galactosidase compared with *amyE* locus over the 48 h. The engineered strain integrating *EclacZ* at *spxA* locus achieved the highest β-galactosidase activity at 1182 U, which was 2.01 times that of the strain using *amyE* locus. The productivity of β-galactosidase using identified integration sites shown the similar trends to these sites using for GFP expression. This result suggested that the identified integration sites could be used for the production of secretary proteins and revealed a consistent expressed trend for both intracellular and extracellular proteins.

## 4 Conclusions

In this study, an integration site library was developed for *B. subtilis*. All integration sites were selected at the 3′-UTR of two opposite nonessential genes to avoid the destruction for the promoter or CDS of endogenous genes, and separated by essential genes to maintain stability when multiple sites were used simultaneously. 12 integration sites revealed higher gene expression strength and lower influence on cell growth, the highest gene expression was found in *spxA* locus, 89% higher than *amyE* locus. A 3-fold gene expression range could be achieved by the developed integration site library without the substitution of promoter and RBS, and gene expression range could be further extended to 15.3-fold or 54.6-fold by combining the design for promoter or RBS, respectively. When the integration library was used to construct *B. subtilis* cell factories, the yields of LNT II and lycopene were improved by 95% and 83%, respectively. In addition, a new integrative shuttle vector was constructed to enable the utilization of strong constitutive promoter with high transcription activity in *E. coli* for the production of secretory proteins in *B. subtilis*. Based on the new shuttle vector, the enzymatic activity of secretory β-galactosidase was improved by 101% using identified integration site *spxA* compared with that integrated in *amyE* locus. The developed integration site library could enrich the genetic parts for *B. subtilis* and accelerate the development of plasmid-free *B. subtilis* microbial cell factories with enhanced genetic stability.

## CRediT authorship contribution statement

**Zhen Zong:** Writing – original draft, Validation, Investigation, Formal analysis, Conceptualization. **Yongting Luo:** Methodology, Investigation. **Wan-Qing Yuan:** Methodology, Investigation. **Feng-Ping Xu:** Investigation. **Jia-Jun Zhu:** Investigation. **Wei Chen:** Writing – review & editing, Methodology. **Wen-Wen Zhou:** Writing – review & editing, Validation, Supervision, Project administration, Methodology, Conceptualization.

## Declaration of competing interest

The authors declare that they have no known competing financial interests or personal relationships that could have appeared to influence the work reported in this paper.
