# Supplementary material for: Identification of integration site library for the development of plasmid-free microbial cell factory in Bacillus subtilis
Source: Synth Syst Biotechnol. 2026 Jun 29;15:126–34. doi: 10.1016/j.synbio.2026.04.042 (PMC13330698; doi:10.1016/j.synbio.2026.04.042)
Supplement: Multimedia component 1 [file mmc1.docx]

**Supporting Information**

**Identification of integration site library for the development of plasmid-free** **microbial cell factory in *Bacillus subtilis***

Zhen Zong^a,1^, Yongting Luo^b,1^, Wan-Qing Yuan^a^, Feng-Ping Xu^a^, Jia-Jun Zhu^a^, Wei Chen^a,*^, Wen-Wen Zhou^a,**^

^a^ Institute of Food Bioscience and Technology, College of Biosystems Engineering and Food Science, Zhejiang University, Hangzhou, Zhejiang, 310058, China

^b^ Department of Nutrition and Health, China Agricultural University, Beijing, 100193, China

^1^ These authors contributed equally to this work.

*****Corresponding author:

Wei Chen, Institute of Food Bioscience and Technology, College of Biosystems Engineering and Food Science, Zhejiang University, Hangzhou, Zhejiang, 310058, China. E-mail: zjuchenwei@zju.edu.cn

******Corresponding author:

Wen-Wen Zhou, Institute of Food Bioscience and Technology, College of Biosystems Engineering and Food Science, Zhejiang University, Hangzhou, Zhejiang, 310058, China. E-mail: vivianzhou11@zju.edu.cn

Number of pages: 15

Number of Tables: 2

Number of Figures: 1

**Contents**

Table S1. Primers used in this study S3

Table S2. Plasmids used in this study S9

Figure S1. Sequencing validation results of new shuttle vector-based secretory expression plasmids for the β-galactosidase S14

**Table S1. Primers used in this study**

| **Name** | **Sequence (5’-3’)** |
| --- | --- |
| abrB H1-F | acttacattaattgcgttgcgatctcttgggaggagaat |
| abrB H1-R | tcctcaattatgttccgcaagttatttaaggttttgaagct |
| abrB H2-F | cggtatcgacatggatgagctcatttcttgtacaaaaaa |
| abrB H2-R | gcagacaatatcagcatccttgtgggcacaaaattattgaatg |
| spxA H1-F | acttacattaattgcgttgcgatatgaaaacattcatacttata |
| spxA H1-R | tcctcaattatgttccgcaagttagtttgccaaacgctgtg |
| spxA H2-F | cggtatcgacatggatgagctagatcgtatcatcaaaa |
| spxA H2-R | gcagacaatatcagcatccttgcatggagcatgactatct |
| tatCY H1-F | acttacattaattgcgttgcgtgctgtttttagcgggct |
| tatCY H1-R | tcctcaattatgttccgcaagttattgcccagaagacac |
| tatCY H2-F | cggtatcgacatggatgagctaaaaaatccatttctcatgcg |
| tatCY H2-R | gcagacaatatcagcatccttggatagccggttttttcattg |
| rlmCD H1-F | acttacattaattgcgttgcggcacaaagcgaagatcat |
| rlmCD H1-R | tcctcaattatgttccgcaagttattcttttaattttatcaacacac |
| rlmCD H2-F | cggtatcgacatggatgagcatagtcaaaaatctatgctgatg |
| rlmCD H2-R | gcagacaatatcagcatccttgaatcatacctttagaagact |
| pchA H1-F | acttacattaattgcgttgcgccgccagacaaaacggaa |
| pchA H1-R | tcctcaattatgttccgcaagttacatatatttgatccaca |
| pchA H2-F | cggtatcgacatggatgagcaaagagtctcacccgcat |
| pchA H2-R | gcagacaatatcagcatccttgcaatacttgatgccggtc |
| yybC H1-F | acttacattaattgcgttgcgtctgccttcaaactgcacg |
| yybC H1-R | tcctcaattatgttccgcaagttaaattgcggctgtgagc |
| yybC H2-F | cggtatcgacatggatgagcatagaaaaggagtgagcctgac |
| yybC H2-R | gcagacaatatcagcatccttgtgctgatgctcaaattaggg |
| yneQ H1-F | acttacattaattgcgttgcgttatgcagaaacag |
| yneQ H1-R | tcctcaattatgttccgcaagttacaaatgaaatcgtataag |
| yneQ H2-F | cggtatcgacatggatgagcaaaaagaacgcacatgaag |
| yneQ H2-R | gcagacaatatcagcatccttgcgccaaatttaaaggcgg |
| rsbRB H1-F | acttacattaattgcgttgcgattgaaagagcaggacagaga |
| rsbRB H1-R | tcctcaattatgttccgcaagttattgatgatagtttaatgc |
| rsbRB H2-F | cggtatcgacatggatgagcaaaaatccgctatctgtgat |
| rsbRB H2-R | gcagacaatatcagcatccttgtccggcatgtttttatatc |
| yhdI H1-F | acttacattaattgcgttgcgttatctcatgtatacgactc |
| yhdI H1-R | tcctcaattatgttccgcaagttaataagatgatatcaacggtgcc |
| yhdI H2-F | cggtatcgacatggatgagcataaaaaacaggccccgg |
| yhdI H2-R | gcagacaatatcagcatccttgggggtttcggtgatgg |
| nupG H1-F | acttacattaattgcgttgcggctcacattcggtttaat |
| nupG H1-R | tcctcaattatgttccgcaagctaccacacaaacaggccg |
| nupG H2-F | cggtatcgacatggatgagcaaacattgagccatatccc |
| nupG H2-R | gcagacaatatcagcatccttgacatgctgaaagccgcggg |
| yqgY H1-F | acttacattaattgcgttgcgtgaaagtctggcttcttg |
| yqgY H1-R | tcctcaattatgttccgcaagttaatggcctccgccgcc |
| yqgY H2-F | cggtatcgacatggatgagcatgcagaaagagacaaacctt |
| yqgY H2-R | gcagacaatatcagcatccttgcataaaaaagaaagaggtg |
| ydfD H1-F | acttacattaattgcgttgcggcggggaagggctatctta |
| ydfD H1-R | tcctcaattatgttccgcaagttacctgcttgccaattcttta |
| ydfD H2-F | cggtatcgacatggatgagccttttatatccttaaatggatgt |
| ydfD H2-R | gcagacaatatcagcatccttgcaaacccaagatcatgtat |
| opuD H1-F | acttacattaattgcgttgcgtacagattctatcggacaat |
| opuD H1-R | tcctcaattatgttccgcaagttaatatgcttttttcactcttg |
| opuD H2-F | cggtatcgacatggatgagccaaaaaagatctttccgcg |
| opuD H2-R | gcagacaatatcagcatccttggcgctcatcggagtga |
| ykrA H1-F | acttacattaattgcgttgcggagaaaaaccgtggcggag |
| ykrA H1-R | tcctcaattatgttccgcaagttattttaaaaggcccaa |
| ykrA H2-F | cggtatcgacatggatgagcagaaacggctggcattgtgc |
| ykrA H2-R | gcagacaatatcagcatccttgaccacacagcatgaaattgac |
| psmB H1-F | acttacattaattgcgttgcgtaaaaatgactgacgttc |
| psmB H1-R | tcctcaattatgttccgcaagttatgaagatgacgtaaga |
| psmB H2-F | cggtatcgacatggatgagccatatacagctgtatatgccttt |
| psmB H2-R | gcagacaatatcagcatccttgcaaacaacaggaaagacaatggtttggc |
| ydbO H1-F | acttacattaattgcgttgcggataggattgcggatttc |
| ydbO H1-R | tcctcaattatgttccgcaagttattttatgtcgtcaggct |
| ydbO H2-F | cggtatcgacatggatgagcggctgtttaacgaattagc |
| ydbO H2-R | gcagacaatatcagcatccttgctgcctccttgctcaaat |
| aldX H1-F | acttacattaattgcgttgcgtgagcttggcggcaagag |
| aldX H1-R | tcctcaattatgttccgcaagttattcagcggcctgaat |
| aldX H2-F | cggtatcgacatggatgagctcgcatgatcaaaaggcgctt |
| aldX H2-R | gcagacaatatcagcatccttgattgaccccagagcaaat |
| racA H1-F | acttacattaattgcgttgcgccattagaactgtatgaacaa |
| racA H1-R | tcctcaattatgttccgcaagttaggtttgaaatttgaacagtg |
| racA H2-F | cggtatcgacatggatgagcagcaaaaagctcccttaaagg |
| racA H2-R | gcagacaatatcagcatccttgatctgtattttctttgaactct |
| ymaD H1-F | acttacattaattgcgttgcgcgaaaacattaataaccaag |
| ymaD H1-R | tcctcaattatgttccgcaagttattctccacccagtttc |
| ymaD H2-F | cggtatcgacatggatgagcagaaaacagacggtcact |
| ymaD H2-R | gcagacaatatcagcatccttgtgatgttgataggatatatattcct |
| hlpB H1-F | acttacattaattgcgttgcgcgctatgccatcgaagta |
| hlpB H1-R | tcctcaattatgttccgcaagtcattttttcttttttcgt |
| hlpB H2-F | cggtatcgacatggatgagcctaaaaagcagccctctc |
| hlpB H2-R | gcagacaatatcagcatccttgtgcgccagaataatgctt |
| gerBC H1-F | acttacattaattgcgttgcgaaaaggaaaccccaatcagca |
| gerBC H1-R | tcctcaattatgttccgcaagttactttcccaccgtgcc |
| gerBC H2-F | cggtatcgacatggatgagcgcaatcaaaagggtgcgcat |
| gerBC H2-R | gcagacaatatcagcatccttggcgcccagcgcttattgc |
| pdaC H1-F | acttacattaattgcgttgcgtcttttgaaagacattctg |
| pdaC H1-R | cctcaattatgttccgcaagttatttcgcttctctttgt |
| pdaC H2-F | cggtatcgacatggatgagcattagaaaaggctgtccg |
| pdaC H2-R | gcagacaatatcagcatccttggatcaaaccttaaagatgat |
| mtnD H1-F | acttacattaattgcgttgcgaagattcaggagctgttctg |
| mtnD H1-R | tcctcaattatgttccgcaagttattgattcacgctgtct |
| mtnD H2-F | cggtatcgacatggatgagcgcgtgagatagccccgttca |
| mtnD H2-R | gcagacaatatcagcatccttgattaaacaatccagctcct |
| ilvE H1-F | acttacattaattgcgttgcgagattgggttccaaagga |
| ilvE H1-R | tcctcaattatgttccgcaagtcacacttccactgtcca |
| ilvE H2-F | cggtatcgacatggatgagcaaatcgaaaaagaacctgccc |
| ilvE H2-R | gcagacaatatcagcatccttgtctctcgctcttctttgt |
| spoVM H1-F | acttacattaattgcgttgcgaaggcaccgtcactctct |
| spoVM H1-R | tcctcaattatgttccgcaagttaatcttttctaaatgagcc |
| spoVM H2-F | cggtatcgacatggatgagctgcccaggggttcaaagcc |
| spoVM H2-R | gcagacaatatcagcatccttgaattctctttccgaagga |
| rlmI H1-F | acttacattaattgcgttgcgacaatacgttgaagatgatgac |
| rlmI H1-R | tcctcaattatgttccgcaagttattttttctgcagaagga |
| rlmI H2-F | cggtatcgacatggatgagcatgaaaaagctgccctgt |
| rlmI H2-R | gcagacaatatcagcatccttgttatgagattcgcccaaa |
| yfkD H1-F | acttacattaattgcgttgcgtcctaattctgtcgcaagc |
| yfkD H1-R | tcctcaattatgttccgcaagttatcttggcagtgagga |
| yfkD H2-F | cggtatcgacatggatgagcataaaaaactgattccaactcg |
| yfkD H2-R | gcagacaatatcagcatccttgtcacccagtatacggcaa |
| cotN H1-F | acttacattaattgcgttgcgaacagcccgtcttcacga |
| cotN H1-R | tcctcaattatgttccgcaagttaagacgtggactcgtt |
| cotN H2-F | cggtatcgacatggatgagctggcgcggacttgtttgttt |
| cotN H2-R | gcagacaatatcagcatccttgccggatcatatttcgtcg |
| yoaH H1-F | acttacattaattgcgttgcgggatctttcttcttcgat |
| yoaH H1-R | tcctcaattatgttccgcaagttaatatttaaaacgattaatgacggt |
| yoaH H2-F | cggtatcgacatggatgagcacaggctacttacgtgca |
| yoaH H2-R | gcagacaatatcagcatccttgacgctggctgaaaacgcc |
| kduD H1-F | acttacattaattgcgttgcgagggatcggtcaaggaatagc |
| kduD H1-R | tcctcaattatgttccgcaagtcagcgggatagccagccg |
| kduD H2-F | cggtatcgacatggatgagccaaataaaaaagctgacgaac |
| kduD H2-R | gcagacaatatcagcatccttgatcttttcttctgctacg |
| fhuD H1-F | acttacattaattgcgttgcgtgagaatgtatttaaaaaccc |
| fhuD H1-R | tcctcaattatgttccgcaagttatttagtcaagctttcc |
| fhuD H2-F | cggtatcgacatggatgagcacaaaagagccgcctgcc |
| fhuD H2-R | gcagacaatatcagcatccttggctcggctggattttgcttg |
| rsbRC H1-F | acttacattaattgcgttgcgtcttgcctttaactataaaata |
| rsbRC H1-R | tcctcaattatgttccgcaagttataccttttttaattttaagtccg |
| rsbRC H2-F | cggtatcgacatggatgagcggcttaaggccttatacc |
| rsbRC H2-R | gcagacaatatcagcatccttgtgaataataatgaaagaacatg |
| guaC H1-F | acttacattaattgcgttgcgcaaagatgaggagtatgaatttgt |
| guaC H1-R | tcctcaattatgttccgcaagttaatatttatccccattaaaaatgg |
| guaC H2-F | cggtatcgacatggatgagctcataaaaaaacgccaagtcag |
| guaC H2-F | gcagacaatatcagcatccttgatgatgtatattccaaaatat |
| ytvI H1-F | acttacattaattgcgttgcgcgtcactcatatccagac |
| ytvI H1-R | tcctcaattatgttccgcaagttatttttgctgaacggt |
| ytvI H2-F | cggtatcgacatggatgagcaacggcagccgttttttcatgg |
| ytvI H2-R | gcagacaatatcagcatccttgcgtctgctcttattacagaag |
| ninI H1-F | acttacattaattgcgttgcgagcatattaaggatgcgat |
| ninI H1-R | tcctcaattatgttccgcaagttacacagaaacagcatc |
| ninI H2-F | cggtatcgacatggatgagctgaaaaacccctgagaga |
| ninI H2-R | gcagacaatatcagcatccttggtattgctggggaaccta |
| ansR H1-F | acttacattaattgcgttgcgattggtgctgtctatattca |
| ansR H1-R | tcctcaattatgttccgcaagttaactcagttcctcctgt |
| ansR H2-F | cggtatcgacatggatgagctctttagctcacggttta |
| ansR H2-R | gcagacaatatcagcatccttgtctttacgccgcataaaa |
| lacA H1-F | acttacattaattgcgttgcgatgtcaaagcttgaaaaaacgca |
| lacA H1-R | tcctcaattatgttccgcaagtcacagtggcaatctcccccg |
| lacA H2-F | cggtatcgacatggatgagcttcaagctatatttggagttg |
| lacA H2-R | gcagacaatatcagcatccttgctaatgtgtgtttacgacaa |
| thrC H1-F | acttacattaattgcgttgcgtgtggaaaggacttatcca |
| thrC H1-R | tcctcaattatgttccgcaagcgaaggcagcagttttttg |
| thrC H2-F | cggtatcgacatggatgagcgtgtgcgaacagctgggg |
| thrC H2-R | gcagacaatatcagcatccttgtcggcaatgtgacaggct |
| sacA H1-F | acttacattaattgcgttgcgacagcacatgaccagga |
| sacA H1-R | tcctcaattatgttccgcaagttccggaaggcgggcgac |
| sacA H2-F | cggtatcgacatggatgagccatccgaccattgactgc |
| sacA H2-R | gcagacaatatcagcatccttgaagacctcttctccgtta |
| gltA H1-F | acttacattaattgcgttgcgaagaacgttacggggtag |
| gltA H1-R | tcctcaattatgttccgcaagcaaatagcggttaggatgc |
| gltA H2-F | cggtatcgacatggatgagcccaaacgctttgttccag |
| gltA H2-R | gcagacaatatcagcatccttggcttctgcaaggccaagc |
| pyrD H1-F | acttacattaattgcgttgcggaggtgaaattgccggga |
| pyrD H1-R | tcctcaattatgttccgcaaggcgattccgcctgttttc |
| pyrD H2-F | cggtatcgacatggatgagcgaagctatccccgaacgt |
| pyrD H2-R | gcagacaatatcagcatccttgatgattccagctccttcc |
| pDG1662-RBS0-GFP-loxp-CmR bb-F | gattaactaataaggaggacaaacatgagtaaaggagaagaactt |
| pDG1662-RBS0-GFP-loxp-CmR bb-R | ttacacagaaacagcatcaataatataagtgtc |
| Pzwf-F | tgatgctgtttctgtgtaaacattttttcagcattctgc |
| Pzwf-R | gtcctccttattagttaatcccttcattttaaatctaaaattcaacg |
| PlepA-F | tgatgctgtttctgtgtaaagtcaatgtatgaatggatacg |
| PlepA-R | gtcctccttattagttaatcactattaaacgcaaaataca |
| pDG1662-Pveg-RBS4-GFP-loxp-CmR part1-F | acttcctttcatgtcaacatttattgtacaacacgagc |
| pDG1662-Pveg-RBS4/RBS7-GFP-loxp-CmR part1-R | caggcatttgagaagcacacg |
| pDG1662-Pveg-RBS4-GFP-loxp-CmR part2-F | tgacatgaaaggaagtatttgaaaatgagtaaaggagaagaacttttcactggag |
| pDG1662-Pveg-RBS4/RBS7-GFP-loxp-CmR part2-R | gtgtgcttctcaaatgcctgaacgccagcaacgcggcc |
| pDG1662-Pveg-RBS7-GFP-loxp-CmR part1-F | agttcctccttcccaccacatttattgtacaacacgagc |
| pDG1662-Pveg-RBS7-GFP-loxp-CmR part2-F | ggtgggaaggaggaactactatgagtaaaggagaagaacttttcactggag |
| Pveg-GFP-rrnBT1T2-Cm^R^-F | cttgcggaacataattgaggaat |
| Pveg-GFP-rrnBT1T2-Cm^R^-R | gctcatccatgtcgataccg |
| pDG1662 backbone-F | caaggatgctgatattgtctgca |
| pDG1662 backbone-R | cgcaacgcaattaatgtaagt |
| Pveg-RBS0-BsaI-rrnB T1-T2 Terminator-Cm^R^-F | cttgcggaacataattgaggaat |
| Pveg-RBS0-BsaI-rrnB T1-T2 Terminator-Cm^R^-R | gctcatccatgtcgataccg |
| pDG1662-spxA-backbone-F | tcctcaattatgttccgcaagttagtttgccaaacgctgtg |
| pDG1662-spxA-backbone-R | cggtatcgacatggatgagctagatcgtatcatcaaaa |
| pDG1662-abrB-backbone-F | tcctcaattatgttccgcaagttatttaaggttttgaagct |
| pDG1662-abrB-backbone-R | cggtatcgacatggatgagctcatttcttgtacaaaaaa |
| pDG1662-guaC-backbone-F | tcctcaattatgttccgcaagttaatatttatccccattaaaaatgg |
| pDG1662-guaC-backbone-R | cggtatcgacatggatgagctcataaaaaaacgccaagtcag |
| pDG1662-ydfD-backbone-F | TCCTCAATTATGTTCCGCAAGttacctgcttgccaattcttta |
| pDG1662-ydfD-backbone-R | cggtaTCGACATGGATGAGCcttttatatccttaaatggatgt |
| pDG1662-psmB-backbone-F | TCCTCAATTATGTTCCGCAAGttatgaagatgacgtaaga |
| pDG1662-psmB-backbone-R | cggtaTCGACATGGATGAGCcatatacagctgtatatgccttt |
| pDG1662-aldX-backbone-F | TCCTCAATTATGTTCCGCAAGttattcagcggcctgaat |
| pDG1662-aldX-backbone-R | cggtaTCGACATGGATGAGCtcgcatgatcaaaaggcgctt |
| pDG1662-tatCY-backbone-F | TCCTCAATTATGTTCCGCAAGttattgcccagaagacac |
| pDG1662-tatCY-backbone-R | cggtaTCGACATGGATGAGCtaaaaaatccatttctcatgcg |
| pSC101 ori-ts-F | ccgggcgtgagactgcggcgaaacagacgaagaatccatggg |
| pSC101 ori-ts-R | gcactgaaatctagagcggtactgtcagaccaagtttactc |
| SpeR-F | cgccgcagtctcacgccc |
| SpeR-R | caggcatttgagaagcacacg |
| pDG1662-Pveg-RBS0-rrnB T1T2 backbone (abrB/amyE/ tatCY/spxA locus) for new shuttle vector-F | accgctctagatttcagtgca |
| pDG1662-Pveg-RBS0-rrnB T1T2 backbone (abrB/amyE/ tatCY/spxA locus) for new shuttle vector-R | gtgtgcttctcaaatgcctgggtttgctccggcgcaaa |
| *EclacY*-F | gattaactaataaggaggacaaacatgtactatttaaaaaacacaaacttttgga |
| *EclacY*-R | gttttatttgatgcggtctccttaagcgacttcattcacctga |
| *NmlgtA*-F | gattaactaataaggaggacaaacatgccgtctgaagcattt |
| *NmlgtA*-R | gttttatttgatgcggtctccttatctatttttcagcagtctatgc |
| *PacrtE*-F | gtgattaactaataaggaggacaaacatgacagtttgcgcgaaaaa |
| *PacrtE*-R | gttttatttgatgcggtctccttagctaactgctgccagtt |
| *PagcrtB*-F | gtgattaactaataaggaggacaaacatgagccaaccgccgctg |
| *PagcrtB*-R | cgttttatttgatgcggtctccttaaaccgggcgttgccac |
| *BtcrtI*-F | gattaactaataaggaggacaaacatgagtgatcagaagaaacatatagtagtg |
| *BtcrtI*-R | cgttttatttgatgcggtctccttaaattctaatatcattgctattctgtcct |
| SPphoB-F | gattaactaataaggaggacaaacatgaaaaaattcccgaagaaattactgc |
| SPphoB-R | cggccagtgaatccgtaatcatggtcatggcgctggcttcaggcac |
| EclacZ-F | accatgattacggattcactgg |
| EclacZ-R | gttttatttgatgcggtctccttatttttgacaccagaccaactggta |

**Table S2. Plasmids used in this study**

| **Name** | | **Description/Sequence** | **Resource** |
| --- | --- | --- | --- |
| pDG1662-loxp-Pveg-RBS0-GFP-rrnBT1T2-*amyE* | | pCloDF13 ori, lox71-Cm^R^-lox66, SpcR for *E. coli* and *B. subtilis*, *amyE* integration site | Lab stock |
| pKD46 | | AmpR, pSC101 ori-repA101(Ts), ParaB-Red | ^[1]^ |
| pDG1662-loxp-Pveg-RBS0-GFP-rrnBT1T2-*spxA* | | pCloDF13 ori, lox71-Cm^R^-lox66, SpcR for *E. coli* and *B. subtilis*, *spxA* integration site | This study |
| pDG1662-loxp-Pveg-RBS0-GFP-rrnBT1T2-*tatCY* | | pCloDF13 ori, lox71-Cm^R^-lox66, SpcR for *E. coli* and *B. subtilis*, *tatCY* integration site | This study |
| pDG1662-loxp-Pveg-RBS0-GFP-rrnBT1T2-*rlmCD* | | pCloDF13 ori, lox71-Cm^R^-lox66, SpcR for *E. coli* and *B. subtilis*, *rlmCD* integration site | This study |
| pDG1662-loxp-Pveg-RBS0-GFP-rrnBT1T2-*abrB* | | pCloDF13 ori, lox71-Cm^R^-lox66, SpcR for *E. coli* and *B. subtilis*, *abrB* integration site | This study |
| pDG1662-loxp-Pveg-RBS0-GFP-rrnBT1T2-*pchA* | | pCloDF13 ori, lox71-Cm^R^-lox66, SpcR for *E. coli* and *B. subtilis*, *pchA* integration site | This study |
| pDG1662-loxp-Pveg-RBS0-GFP-rrnBT1T2-*yybC* | | pCloDF13 ori, lox71-Cm^R^-lox66, SpcR for *E. coli* and *B. subtilis*, *yybC* integration site | This study |
| pDG1662-loxp-Pveg-RBS0-GFP-rrnBT1T2-*yneQ* | | pCloDF13 ori, lox71-Cm^R^-lox66, SpcR for *E. coli* and *B. subtilis*, *yneQ* integration site | This study |
| pDG1662-loxp-Pveg-RBS0-GFP-rrnBT1T2-*rsbRB* | | pCloDF13 ori, lox71-Cm^R^-lox66, SpcR for *E. coli* and *B. subtilis*, *rsbRB* integration site | This study |
| pDG1662-loxp-Pveg-RBS0-GFP-rrnBT1T2-*yhdI* | | pCloDF13 ori, lox71-Cm^R^-lox66, SpcR for *E. coli* and *B. subtilis*, *yhdI* integration site | This study |
| pDG1662-loxp-Pveg-RBS0-GFP-rrnBT1T2-*nupG* | | pCloDF13 ori, lox71-Cm^R^-lox66, SpcR for *E. coli* and *B. subtilis*, *nupG* integration site | This study |
| pDG1662-loxp-Pveg-RBS0-GFP-rrnBT1T2-*yqgY* | | pCloDF13 ori, lox71-Cm^R^-lox66, SpcR for *E. coli* and *B. subtilis*, *yqgY* integration site | This study |
| pDG1662-loxp-Pveg-RBS0-GFP-rrnBT1T2-*ydfD* | | pCloDF13 ori, lox71-Cm^R^-lox66, SpcR for *E. coli* and *B. subtilis*, *ydfD* integration site | This study |
| pDG1662-loxp-Pveg-RBS0-GFP-rrnBT1T2-*opuD* | | pCloDF13 ori, lox71-Cm^R^-lox66, SpcR for *E. coli* and *B. subtilis*, *opuD* integration site | This study |
| pDG1662-loxp-Pveg-RBS0-GFP-rrnBT1T2-*ykrA* | | pCloDF13 ori, lox71-Cm^R^-lox66, SpcR for *E. coli* and *B. subtilis*, *ykrA* integration site | This study |
| pDG1662-loxp-Pveg-RBS0-GFP-rrnBT1T2-*psmB* | | pCloDF13 ori, lox71-Cm^R^-lox66, SpcR for *E. coli* and *B. subtilis*, *psmB* integration site | This study |
| pDG1662-loxp-Pveg-RBS0-GFP-rrnBT1T2-*ydbO* | | pCloDF13 ori, lox71-Cm^R^-lox66, SpcR for *E. coli* and *B. subtilis*, *ydbO* integration site | This study |
| pDG1662-loxp-Pveg-RBS0-GFP-rrnBT1T2-*aldX* | | pCloDF13 ori, lox71-Cm^R^-lox66, SpcR for *E. coli* and *B. subtilis*, *aldX* integration site | This study |
| pDG1662-loxp-Pveg-RBS0-GFP-rrnBT1T2-*racA* | | pCloDF13 ori, lox71-Cm^R^-lox66, SpcR for *E. coli* and *B. subtilis*, *racA* integration site | This study |
| pDG1662-loxp-Pveg-RBS0-GFP-rrnBT1T2-*ymaD* | | pCloDF13 ori, lox71-Cm^R^-lox66, SpcR for *E. coli* and *B. subtilis*, *ymaD* integration site | This study |
| pDG1662-loxp-Pveg-RBS0-GFP-rrnBT1T2-*hlpB* | | pCloDF13 ori, lox71-Cm^R^-lox66, SpcR for *E. coli* and *B. subtilis*, *hlpB* integration site | This study |
| pDG1662-loxp-Pveg-RBS0-GFP-rrnBT1T2-*gerBC* | | pCloDF13 ori, lox71-Cm^R^-lox66, SpcR for *E. coli* and *B. subtilis*, *gerBC* integration site | This study |
| pDG1662-loxp-Pveg-RBS0-GFP-rrnBT1T2-*pdaC* | | pCloDF13 ori, lox71-Cm^R^-lox66, SpcR for *E. coli* and *B. subtilis*, *pdaC* integration site | This study |
| pDG1662-loxp-Pveg-RBS0-GFP-rrnBT1T2-*mtnD* | | pCloDF13 ori, lox71-Cm^R^-lox66, SpcR for *E. coli* and *B. subtilis*, *mtnD* integration site | This study |
| pDG1662-loxp-Pveg-RBS0-GFP-rrnBT1T2-*ilvE* | | pCloDF13 ori, lox71-Cm^R^-lox66, SpcR for *E. coli* and *B. subtilis*, *ilvE* integration site | This study |
| pDG1662-loxp-Pveg-RBS0-GFP-rrnBT1T2-*spoVM* | | pCloDF13 ori, lox71-Cm^R^-lox66, SpcR for *E. coli* and *B. subtilis*, *spoVM* integration site | This study |
| pDG1662-loxp-Pveg-RBS0-GFP-rrnBT1T2-*rlmI* | | pCloDF13 ori, lox71-Cm^R^-lox66, SpcR for *E. coli* and *B. subtilis*, *rlmI* integration site | This study |
| pDG1662-loxp-Pveg-RBS0-GFP-rrnBT1T2-*yfkD* | | pCloDF13 ori, lox71-Cm^R^-lox66, SpcR for *E. coli* and *B. subtilis*, *yfkD* integration site | This study |
| pDG1662-loxp-Pveg-RBS0-GFP-rrnBT1T2-*cotN* | | pCloDF13 ori, lox71-Cm^R^-lox66, SpcR for *E. coli* and *B. subtilis*, *cotN* integration site | This study |
| pDG1662-loxp-Pveg-RBS0-GFP-rrnBT1T2-*yoaH* | | pCloDF13 ori, lox71-Cm^R^-lox66, SpcR for *E. coli* and *B. subtilis*, *yoaH* integration site | This study |
| pDG1662-loxp-Pveg-RBS0-GFP-rrnBT1T2-*kduD* | | pCloDF13 ori, lox71-Cm^R^-lox66, SpcR for *E. coli* and *B. subtilis*, *kduD* integration site | This study |
| pDG1662-loxp-Pveg-RBS0-GFP-rrnBT1T2-*fhuD* | | pCloDF13 ori, lox71-Cm^R^-lox66, SpcR for *E. coli* and *B. subtilis*, *fhuD* integration site | This study |
| pDG1662-loxp-Pveg-RBS0-GFP-rrnBT1T2-*rsbRC* | | pCloDF13 ori, lox71-Cm^R^-lox66, SpcR for *E. coli* and *B. subtilis*, *rsbRC* integration site | This study |
| pDG1662-loxp-Pveg-RBS0-GFP-rrnBT1T2-*guaC* | | pCloDF13 ori, lox71-Cm^R^-lox66, SpcR for *E. coli* and *B. subtilis*, *guaC* integration site | This study |
| pDG1662-loxp-Pveg-RBS0-GFP-rrnBT1T2-*ytvI* | | pCloDF13 ori, lox71-Cm^R^-lox66, SpcR for *E. coli* and *B. subtilis*, *ytvI* integration site | This study |
| pDG1662-loxp-Pveg-RBS0-GFP-rrnBT1T2-*ninI* | | pCloDF13 ori, lox71-Cm^R^-lox66, SpcR for *E. coli* and *B. subtilis*, *ninI* integration site | This study |
| pDG1662-loxp-Pveg-RBS0-GFP-rrnBT1T2-*ansR* | | pCloDF13 ori, lox71-Cm^R^-lox66, SpcR for *E. coli* and *B. subtilis*, *ansR* integration site | This study |
| pDG1662-loxp-Pveg-RBS0-GFP-rrnBT1T2-*lacA* | | pCloDF13 ori, lox71-Cm^R^-lox66, SpcR for *E. coli* and *B. subtilis*, *lacA* integration site | This study |
| pDG1662-loxp-Pveg-RBS0-GFP-rrnBT1T2-*thrC* | | pCloDF13 ori, lox71-Cm^R^-lox66, SpcR for *E. coli* and *B. subtilis*, *thrC* integration site | This study |
| pDG1662-loxp-Pveg-RBS0-GFP-rrnBT1T2-*sacA* | | pCloDF13 ori, lox71-Cm^R^-lox66, SpcR for *E. coli* and *B. subtilis*, *sacA* integration site | This study |
| pDG1662-loxp-Pveg-RBS0-GFP-rrnBT1T2-*gltA* | | pCloDF13 ori, lox71-Cm^R^-lox66, SpcR for *E. coli* and *B. subtilis*, *gltA* integration site | This study |
| pDG1662-loxp-Pveg-RBS0-GFP-rrnBT1T2-*pyrD* | | pCloDF13 ori, lox71-Cm^R^-lox66, SpcR for *E. coli* and *B. subtilis*, *pyrD* integration site | This study |
| pDG1662-loxp-Pzwf-RBS0-GFP-rrnBT1T2-*ninI* | | pCloDF13 ori, lox71-Cm^R^-lox66, SpcR for *E. coli* and *B. subtilis*, *ninI* integration site | This study |
| pDG1662-loxp-PlepA-RBS0-GFP-rrnBT1T2-*ninI* | | pCloDF13 ori, lox71-Cm^R^-lox66, SpcR for *E. coli* and *B. subtilis*, *ninI* integration site | This study |
| pDG1662-loxp-Pveg-RBS4-GFP-rrnBT1T2-*ninI* | | pCloDF13 ori, lox71-Cm^R^-lox66, SpcR for *E. coli* and *B. subtilis*, *ninI* integration site | This study |
| pDG1662-loxp-Pveg-RBS7-GFP-rrnBT1T2-*ninI* | | pCloDF13 ori, lox71-Cm^R^-lox66, SpcR for *E. coli* and *B. subtilis*, *ninI* integration site | This study |
| pDG1662-loxp-Pveg-RBS0-BsaI-rrnBT1T2-*amyE* | | pCloDF13 ori, lox71-Cm^R^-lox66, SpcR for *E. coli* and *B. subtilis*, *amyE* integration site | Lab stock |
| pDG1662-loxp-Pveg-RBS0-BsaI-rrnBT1T2-*guaC* | | pCloDF13 ori, lox71-Cm^R^-lox66, SpcR for *E. coli* and *B. subtilis*, *guaC* integration site | This study |
| pDG1662-loxp-Pveg-RBS0-BsaI-rrnBT1T2-*abrB* | | pCloDF13 ori, lox71-Cm^R^-lox66, SpcR for *E. coli* and *B. subtilis*, *abrB* integration site | This study |
| pDG1662-loxp-Pveg-RBS0-BsaI-rrnBT1T2-*spxA* | | pCloDF13 ori, lox71-Cm^R^-lox66, SpcR for *E. coli* and *B. subtilis*, *spxA* integration site | This study |
| pDG1662-loxp-Pveg-RBS0-BsaI-rrnBT1T2-*tatCY* | | pCloDF13 ori, lox71-Cm^R^-lox66, SpcR for *E. coli* and *B. subtilis*, *tatCY* integration site | This study |
| pDG1662-loxp-Pveg-RBS0-BsaI-rrnBT1T2-*ydfD* | | pCloDF13 ori, lox71-Cm^R^-lox66, SpcR for *E. coli* and *B. subtilis*, *ydfD* integration site | This study |
| pDG1662-loxp-Pveg-RBS0-BsaI-rrnBT1T2-*psmB* | | pCloDF13 ori, lox71-Cm^R^-lox66, SpcR for *E. coli* and *B. subtilis*, *psmB* integration site | This study |
| pDG1662-loxp-Pveg-RBS0-BsaI-rrnBT1T2-*aldX* | | pCloDF13 ori, lox71-Cm^R^-lox66, SpcR for *E. coli* and *B. subtilis*, *aldX* integration site | This study |
| pDG1662-loxp-Pveg-RBS0-*NmlgtA*-rrnBT1T2-*amyE* | | Integrating *NmlgtA* in *amyE* locus. | This study |
| pDG1662-loxp-Pveg-RBS0-*EclacY*-rrnBT1T2-*guaC* | | Integrating *EclacY* in *guaC* locus. | This study |
| pDG1662-loxp-Pveg-RBS0- *NmlgtA*-rrnBT1T2-*abrB* | | Integrating *NmlgtA* in *abrB* locus. | This study |
| pDG1662-loxp-Pveg-RBS0- *PaCrtE*-rrnBT1T2-*spxA* | | Integrating *PaCrtE* in *spxA* locus. | This study |
| pDG1662-loxp-Pveg-RBS0- *PagCrtB*-rrnBT1T2-*tatCY* | | Integrating *PagCrtB* in *tatCY* locus. | This study |
| pDG1662-loxp-Pveg-RBS0- *BtCrtI*-rrnBT1T2-*abrB* | | Integrating *BtCrtI* in *abrB* locus. | This study |
| pDG1662-loxp-Pveg-RBS0- *PaCrtE*-rrnBT1T2-*ydfD* | | Integrating *PaCrtE* in *ydfD* locus. | This study |
| pDG1662-loxp-Pveg-RBS0- *PagCrtB*-rrnBT1T2-*psmB* | | Integrating *PagCrtB* in *psmB* locus. | This study |
| pDG1662-loxp-Pveg-RBS0- *BtCrtI*-rrnBT1T2-*aldX* | | Integrating *BtCrtI* in *aldX* locus. | This study |
| pBSSecreation-loxp-Pveg-RBS0-BsaI-rrnBT1T2-*amyE* | | pSC101-ts replicon, lox71-Cm^R^-lox66, SpcR for *E. coli*, *amyE* integration site | This study |
| pBSSecreation-loxp-Pveg-RBS0-BsaI-rrnBT1T2-*spxA* | | pSC101-ts replicon, lox71-Cm^R^-lox66, SpcR for *E. coli*, *spxA* integration site | This study |
| pBSSecreation-loxp-Pveg-RBS0-BsaI-rrnBT1T2-*tatCY* | | pSC101-ts replicon, lox71-Cm^R^-lox66, SpcR for *E. coli*, *tatCY* integration site | This study |
| pBSSecreation-loxp-Pveg-RBS0-BsaI-rrnBT1T2-*abrB* | | pSC101-ts replicon, lox71-Cm^R^-lox66, SpcR for *E. coli*, *abrB* integration site | This study |
| pBSSecreation-loxp-Pveg-RBS0-SPphoB-*EclacZ*-rrnBT1T2-*amyE* | | Integrating SPphoB-*EclacZ* in *amyE* locus. | This study |
| pBSSecreation-loxp-Pveg-RBS0-SPphoB-*EclacZ*-rrnBT1T2-*spxA* | | Integrating SPphoB-*EclacZ* in *spxA* locus. | This study |
| pBSSecreation-loxp-Pveg-RBS0-SPphoB-*EclacZ*-rrnBT1T2-*tatCY* | | Integrating SPphoB-*EclacZ* in *tatCY* locus. | This study |
| pBSSecreation-loxp-Pveg-RBS0-SPphoB-*EclacZ*-rrnBT1T2-*abrB* | | Integrating SPphoB-*EclacZ* in *abrB* locus. | This study |
| pDG1662-loxp-Pveg-RBS0-GFP-rrnBT1T2-*amyE* | ggagaccgcatcaaataaaacgaaaggctcagtcgaaagactgggcctttcgttttatctgttgtttgtcggtgaacgctctcctgagtaggacaaatccgccgctctagctaagcagaaggccatcctgacggatggcctttttgcctagtaccgttcgtatagcatacattatacgaagttattgacagcttatcatcggcaatagttacccttattatcaagataagaaagaaaaggatttttcgctacgctcaaatcctttaaaaaaacacaaaagaccacattttttaatgtggtcttttattcttcaactaaagcacccattagttcaacaaacgaaaattggataaagtgggatatttttaaaatatatatttatgttacagtaatattgacttttaaaaaaggattgattctaatgaagaaagcagacaagtaagcctcctaaattcactttagataaaaatttaggaggcatatcaaatgaactttaataaaattgatttagacaattggaagagaaaagagatatttaatcattatttgaaccaacaaacgacttttagtataaccacagaaattgatattagtgttttataccgaaacataaaacaagaaggatataaattttaccctgcatttattttcttagtgacaagggtgataaactcaaatacagcttttagaactggttacaatagcgacggagagttaggttattgggataagttagagccactttatacaatttttgatggtgtatctaaaacattctctggtatttggactcctgtaaagaatgacttcaaagagttttatgatttatacctttctgatgtagagaaatataatggttcggggaaattgtttcccaaaacacctatacctgaaaatgctttttctctttctattattccatggacttcatttactgggtttaacttaaatatcaataataatagtaattaccttctacccattattacagcaggaaaattcattaataaaggtaattcaatatatttaccgctatctttacaggtacatcattctgtttgtgatggttatcatgcaggattgtttatgaactctattcaggaattgtcagataggcctaatgactggcttttataatatgagataatgccgactgtactttttacagtcggttttctaatgtcactaacctgccccgttagttgaagaaggtttttatattacagctccagatcctctacgccggacgcatcgtggccggcatcaccggcgccacaggtgcggttgctggcgcctatatcgccgacatcaccgatggggaagatcgggctcgccacttcgggctcatgagcgcttgtttcggcgtgggtatggtggcaggccccgtggccgggggactgttgggcgccatctccttgcatgcaccattccttgcggcggcggtgctcaacggcctcaacctactactgggctgcttcctaatgcaggagtcgcataagggagagcgataacttcgtatagcatacattatacgaacggtatcgacatggatgagcgatgatgatatccgtttaggctgggcggtgatagcttctcgttcaggcagtacgcctcttttcttttccagacctgagggaggcggaaatggtgtgaggttcccggggaaaagccaaataggcgatcgcgggagtgctttatttgaagatcaggctatcactgcggtcaatagatttcacaatgtgatggctggacagcctgaggaactctcgaacccgaatggaaacaaccagatatttatgaatcagcgcggctcacatggcgttgtgctggcaaatgcaggttcatcctctgtctctatcaatacggcaacaaaattgcctgatggcaggtatgacaataaagctggagcgggttcatttcaagtgaacgatggtaaactgacaggcacgatcaatgccaggtctgtagctgtgctttatcctgatgatattgcaaaagcgcctcatgttttccttgagaattacaaaacaggtgtaacacattctttcaatgatcaactgacgattaccttgcgtgcagatgcgaatacaacaaaagccgtttatcaaatcaataatggaccagacgacaggcgtttaaggatggagatcaattcacaatcggaaaaggagatccaatttggcaaaacatacaccatcatgttaaaaggaacgaacagtgatggtgtaacgaggaccgagaaatacagttttgttaaaagagatccagcgtcggccaaaaccatcggctatcaaaatccgaatcattggagccaggtaaatgcttatatctataaacatgatgggagccgagtaattgaattgaccggatcttggcctggaaaaccaatgactaaaaatgcagacggaatttacacgctgacgctgcctgcggacacggatacaaccaacgcaaaagtgatttttaataatggcagcgcccaagtgcccggtcagaatcagcctggctttgattacgtgctaaatggtttatataatgactcgggcttaagcggttctcttccccattgagggcaaggctagacgggacttaccgaaagaaaccatcaatgatggtttcttttttgttcataaatcagacaaaacttttctcttgcaaaagtttgtgaagtgttgcacaatataaatgtgaaatacttcacaaacaaaaagacatcaaagagaaacataccctgcaaggatgctgatattgtctgcatttgcgccggagcaaaccaaaaacctggtgagacacgccttgaattagtagaaaagaacttgaagattttcaaaggcatcgttagtgaagtcatggcgagcggatttgacggcattttcttagtcgcgacgcgaggctggatggccttccccattatgattcttctcgcttccggcggcatcgggatgcccgcgttgcaggccatgctgtccaggcaggtagatgacgaccatcagggacagcttcaaggatcgctcgcggctcttaccagcctaacttcgatcactggaccgctgatcgtcacggcgatttatgccgcctcggcgagcacatggaacgggttggcatggattgtaggcgccgccctataccttgtctgcctccccgcgttgcgtcgcggtgcatggagccgggccacctactgaagtggatttctttaagagctcctttaacttcctcaccagtagttgtatcggtaccataagtagaagcagcaacccaagtagctttaccagcatccggttcaaccagcatagtaagaatcttactggacatcggcagttcttcgaacagtgcgccaactaccagctctttctgcagttcattcagggcaccggagaacctgcgtgcaatccatcttgttcaatcatgcgaaacgatcctcatcctgtctcttgatccatggattacgcgttaacccgggcccgcggatgcatatgatcagatcttaaggcctaggtctagaggatcgatctgtataataaagaataattattaatctgtagacaaattgtgaaaggatgtacttaaacgctaacggtcagctttattgaacagtaatttaagtatatgtccaatctagggtaagtaaattgagtatcaatataaactttatatgaacataatcaacgaggtgaaatcatgagcaatttgattaacggaaaaataccaaatcaagcgattcaaacattaaaaatcgtaaaagatttatttggaagttcaatagttggagtatatctatttggttcagcagtaaatggtggtttacgcattaacagcgatgtagatgttctagtcgtcgtgaatcatagtttacctcaattaactcgaaaaaaactaacagaaagactaatgactatatcaggaaagattggaaatacggattctgttagaccacttgaagttacggttataaataggagtgaagttgtcccttggcaatatcctccaaaaagagaatttatatacggtgagtggctcaggggtgaatttgagaatggacaaattcaggaaccaagctatgatcctgatttggctattgttttagcacaagcaagaaagaatagtatttctctatttggtcctgattcttcaagtatacttgtctccgtacctttgacagatattcgaagagcaattaaggattctttgccagaactaattgaggggataaaaggtgatgagcgtaatgtaattttaaccctagctcgaatgtggcaaacagtgactactggtgaaattacctcgaaagatgtcgctgcagaatgggctatacctcttttacctaaagagcatgtaactttactggatatagctagaaaaggctatcggggagagtgtgatgataagtgggaaggactatattcaaaggtgaaagcactcgttaagtatatgaaaaattctatagaaacttctctcaattaggctaattttattgcaataacaggtgcttacttttctggagttctttagcaaatttttttattagctgaacttagtattagtggccatactcctccaatccaaagctatttagaaagattactatatcctcaaacaggcggtaaccggcctcttcatcgggaatgcgcgcgaccttcagcatcgccggcatgtccccctggcggacgggaagtatccagctcgaggtcgggccgcgttgctggcgttcaggcatttgagaagcacacggtcacactgcttccggtagtcaataaaccggtaaaccagcaatagacataagcggctatttaacgaccctgccctgaaccgacgaccgggtcatcgtggccggatcttgcggcccctcggcttgaacgaattgttagacattatttgccgactaccttggtgatctcgcctttcacgtagtggacaaattcttccaactgatctgcgcgcgaggccaagcgatcttcttcttgtccaagataagcctgtctagcttcaagtatgacgggctgatactgggccggcaggcgctccattgcccagtcggcagcgacatccttcggcgcgattttgccggttactgcgctgtaccaaatgcgggacaacgtaagcactacatttcgctcatcgccagcccagtcgggcggcgagttccatagcgttaaggtttcatttagcgcctcaaatagatcctgttcaggaaccggatcaaagagttcctccgccgctggacctaccaaggcaacgctatgttctcttgcttttgtcagcaagatagccagatcaatgtcgatcgtggctggctcgaagatacctgcaagaatgtcattgcgctgccattctccaaattgcagttcgcgcttagctggataacgccacggaatgatgtcgtcgtgcacaacaatggtgacttctacagcgcggagaatctcgctctctccaggggaagccgaagtttccaaaaggtcgttgatcaaagctcgccgcgttgtttcatcaagccttacggtcaccgtaaccagcaaatcaatatcactgtgtggcttcaggccgccatccactgcggagccgtacaaatgtacggccagcaacgtcggttcgagatggcgctcgatgacgccaactacctctgatagttgagtcgatacttcggcgatcaccgcttccctcatactcttcctttttcaatattattgaagcatttatcagggttattgtctcatgagcggatacatatttgaatgtatttagaaaaataaacaaatagctagctcactcggtcgctacgctccgggcgtgagactgcggcgggcgctgcggacacatacaaagttacccacagattccgtggataagcaggggactaacatgtgaggcaaaacagcagggccgcgccggtggcgtttttccataggctccgccctcctgccagagttcacataaacagacgcttttccggtgcatctgtgggagccgtgaggctcaaccatgaatctgacagtacgggcgaaacccgacaggacttaaagatccccaccgtttccggcgggtcgctccctcttgcgctctcctgttccgaccctgccgtttaccggatacctgttccgcctttctcccttacgggaagtgtggcgctttctcatagctcacacactggtatctcggctcggtgtaggtcgttcgctccaagctgggctgtaagcaagaactccccgttcagcccgactgctgcgccttatccggtaactgttcacttgagtccaacccggaaaagcacggtaaaacgccactggcagcagccattggtaactgggagttcgcagaggatttgtttagctaaacacgcggttgctcttgaagtgtgcgccaaagtccggctacactggaaggacagatttggttgctgtgctctgcgaaagccagttaccacggttaagcagttccccaactgacttaaccttcgatcaaaccacctccccaggtggttttttcgtttacagggcaaaagattacgcgcagaaaaaaaggatctcaagaagatcctttgatcttttctactgaaccgctctagatttcagtgcaatttatctcttcaaatgtagcacctgaagtcagccccatacgatataagttgtaattctcatgttagtcatgccccgcgcccaccggaaggagctgactgggttgaaggctctcaagggcatcggtcgagatcccggtgcctaatgagtgagctaacttacattaattgcgttgcgcggaagaatgaagtaagagggatttttgactccgaagtaagtcttcaaaaaatcaaataaggagtgtcaagaatgtttgcaaaacgattcaaaacctctttactgccgttattcgctggatttttattgctgtttcatttggttctggcaggaccggcggctgcgagtgctgaaacggcgaacaaatcgaatgagcttacagcaccgtcgatcaaaagcggaaccattcttcatgcatggaattggtcgttcaatacgttaaaacacaatatgaaggatattcatgatgcaggatatacagccattcagacatctccgattaaccaagtaaaggaagggaatcaaggagataaaagcatgtcgaactggtactggctgtatcagccgacatcgtatcaaattggcaaccgttacttaggtactgaacaagaatttaaagaaatgtgtgcagccgctgaagaatatggcataaaggtcattgttgacgcggtcatcaatcataccaccagtgattatgccgcgatttccaatgaggttaagagtattccaaactggacacatggaaacacacaaattaaaaactggtctgatcgcttgcggaacataattgaggaatcatagaattttgtcaaaataattttattgacaacgtcttattaacgttgatataatttaaattttatttgacaaaaatgggctcgtgttgtacaataaatgtgattaactaataaggaggacaaacatgagtaaaggagaagaacttttcactggagttgtcccaattcttgttgaattagatggtgatgttaatgggcacaaattttctgtcagtggagagggtgaaggtgatgcaacatacggaaaacttacccttaaatttatttgcactactggaaaactacctgttccatggccaacacttgtcactactttcggttatggtgttcaatgctttgcgagatacccagatcatatgaaacagcatgactttttcaagagtgccatgcccgaaggttatgtacaggaaagaactatatttttcaaagatgacgggaactacaagacacgtgctgaagtcaagtttgaaggtgatacccttgttaatagaatcgagttaaaaggtattgattttaaagaagatggaaacattcttggacacaaattggaatacaactataactcacacaatgtatacatcatggcagacaaacaaaagaatggaatcaaagttaacttcaaaattagacacaacattgaagatggaagcgttcaactagcagaccattatcaacaaaatactccaattggcgatggccctgtccttttaccagacaaccattacctgtccacacaatctgccctttcgaaagatcccaacgaaaagagagaccacatggtccttcttgagtttgtaacagctgctgggattacacatggcatggatgaactatacaaataa | | |

**Figure S1. Sequencing results of the construction of new shuttle vector-based plasmids for the production of secretory β-galactosidase.**

**References**

1. Datsenko, K. A., and Wanner, B. L. One-step inactivation of chromosomal genes in *Escherichia coli* K-12 using PCR products, Proc Natl Acad Sci U S A 2000; 97(12): 6640-6645. https://doi.org/10.1073/pnas.120163297
